# HDAC10 deletion promotes Foxp3^+^ T-regulatory cell function

**DOI:** 10.1038/s41598-019-57294-x

**Published:** 2020-01-16

**Authors:** Satinder Dahiya, Ulf H. Beier, Liqing Wang, Rongxiang Han, Jing Jiao, Tatiana Akimova, Alessia Angelin, Douglas C. Wallace, Wayne W. Hancock

**Affiliations:** 1 Division of Transplant Immunology, Department of Pathology and Laboratory Medicine, and Biesecker Center for Pediatric Liver Disease, Children’s Hospital of Philadelphia and University of Pennsylvania, Philadelphia, PA 19104 USA; 2 Division of Nephrology and Department of Pediatrics, Children’s Hospital of Philadelphia and University of Pennsylvania, Philadelphia, PA 19104 USA; 3 Center for Mitochondrial and Epigenomic Medicine, Children’s Hospital of Philadelphia, Philadelphia, PA 19104 USA

**Keywords:** Mechanisms of disease, Adaptive immunity, Translational immunology

## Abstract

Foxp3^+^ T-regulatory (Treg) cells are capable of suppressing immune responses. Lysine acetylation is a key mechanism of post-translational control of various transcription factors, and when acetylated, Foxp3 is stabilized and transcriptionally active. Therefore, understanding the roles of various histone/protein deacetylases (HDAC) are key to promoting Treg-based immunotherapy. Several of the 11 classical HDAC enzymes are necessary for optimal Treg function while others are dispensable. We investigated the effect of HDAC10 in murine Tregs. HDAC10 deletion had no adverse effect on the health of mice, which retained normal CD4^+^ and CD8^+^ T cell function. However, HDAC10^−/−^ Treg exhibited increased suppressive function *in vitro* and *in vivo*. C57BL/6 Rag1^−/−^ mice adoptively transferred with HDAC10^−/−^ but not wild Treg, were protected from developing colitis. HDAC10^−/−^ but not wild-type mice receiving fully MHC-mismatched cardiac transplants became tolerant and showed long-term allograft survival (>100 d). We conclude that targeting of HDAC10 may be of therapeutic value for inflammatory disorders including colitis and also for transplantation.

## Introduction

Foxp3^+^ T-regulatory (Treg) cells are a subset of T cells essential for maintaining immune homeostasis and preventing autoimmunity^1^, but can also have adverse effects during chronic infections and by limiting anti-cancer immunity^2^. Augmenting Treg cell function is a promising therapeutic strategy to achieve therapeutic immunosuppression. Foxp3, the key transcription factor of Treg is regulated by various post-translational modifications, including lysine acetylation^3^. In the acetylated state, Foxp3 is stabilized, and, in addition, shows better DNA binding and higher efficiency when acting as a transcription factor^4^. For this reason, histone/protein deacetylase (HDAC) enzymes controlling Foxp3 lysine acetylation are of particular interest for therapeutic immunosuppression^5^. Several HDAC enzymes have been explored: loss of HDAC6, HDAC9 and Sirtuin-1 in each case augments Foxp3 acetylation and Treg function^6–10^, while deletion of HDAC3, HDAC5 or Sirtuin-3 was shown to impair Foxp3^+^ Treg function^11–13^. Among the different HDACs, the class IIb HDAC6, is the most promising candidate for immunosuppressive therapy^5,14^. Global knockout of HDAC6 does not cause overt illness, other than an increase in weight in male mice^15,16^, and specific inhibitors are available with good immunosuppressive properties *in vivo*^8,10^. As HDAC6 inhibitors are being evaluated in clinical trials, it is important to assess the immune phenotype of HDAC10 deletion, since some of the class IIb inhibitors, such as tubastatin A, target both HDAC6 and HDAC10^17^. Compared to other HDAC isoforms, relatively little is known about HDAC10. Since its discovery in 2002^18,19^, HDAC10 has been reported to be involved in DNA repair, autophagy, and cancer^17,20,21^. Like HDAC6, the other class IIb HDAC, HDAC10 has two deacetylase domains, although only one is considered functional^18^. Wang *et al*. have reported a pro-inflammatory phenotype of HDAC10-deficient antigen-presenting cells, with increased priming of T cells^22^. Here, we report a characterization of the HDAC10^−/−^ immune phenotype. We observed, similar to HDAC6-deficient mice, a pro-tolerant phenotype, with increased Foxp3^+^ Treg suppressive function, which translated to improved outcomes in autoimmune colitis and cardiac allograft models. These data provide a rationale for further development of class IIb HDAC isoform-selective targeting.

## Results

### HDAC10 deletion does not affect conventional T cell function

**Figure 1:**
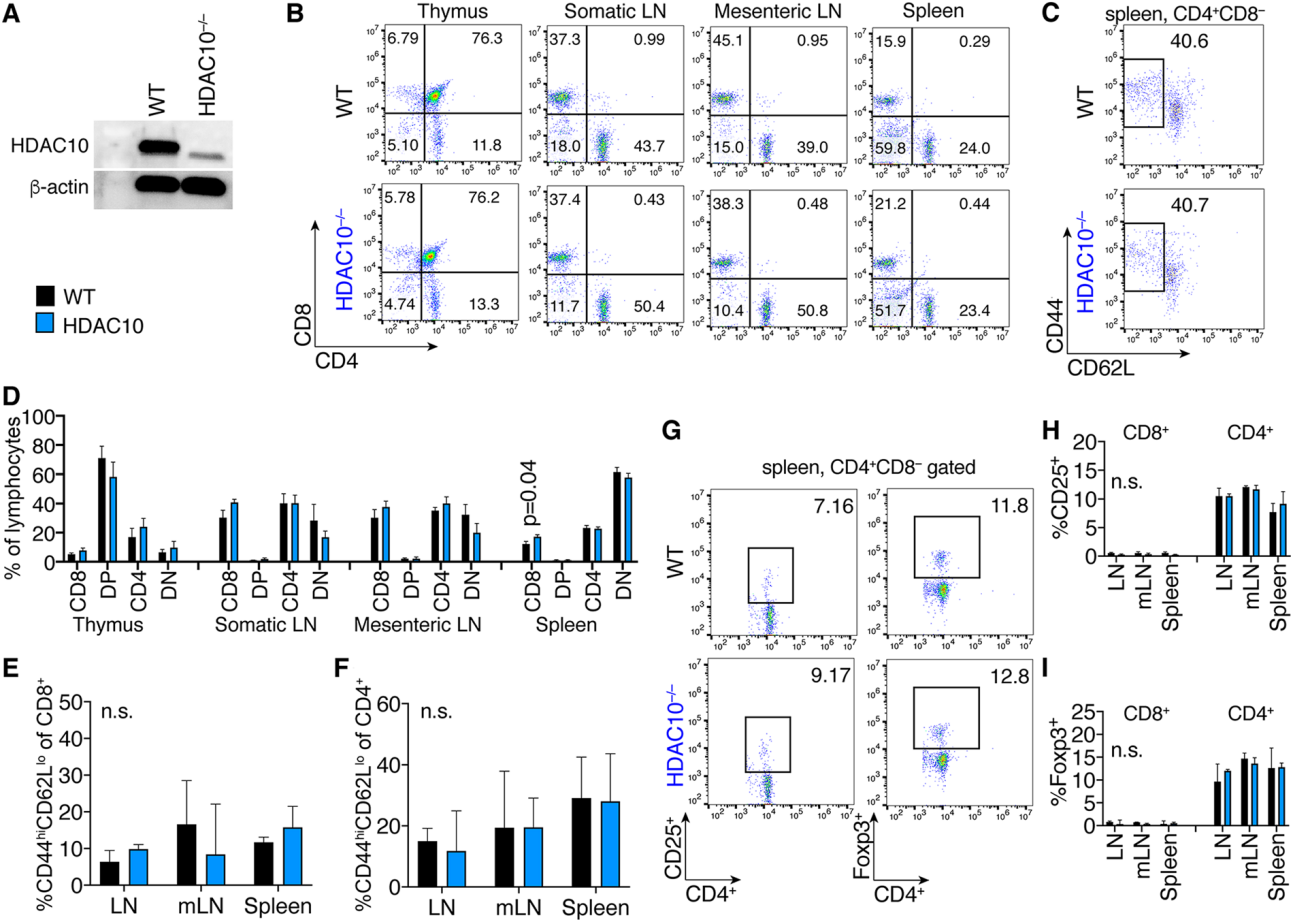
HDAC10 deletion does not substantially affect baseline lymphocyte populations. (**A**) Western blot of splenocytes from C57BL/6 wild type (WT) or HDAC10^−/−^. (**B**,**C**) Representative CD4^+^ and CD8^+^ (**B**), as well as CD4^+^CD44^hi^CD62L^lo^ (C) lymphocyte populations of WT and HDAC10^−/−^ mice. (**D**–**F**) Pooled data from 3–6 (**D**) and 3 (**E**,**F**) independent experiments. (**G**–**I**) Representative (**G**) and pooled data (**H,I**) from three independent experiments. (**H**) CD25^+^ and (**I**) Foxp3^+^ of CD4^+^CD8^−^ T cell populations. Statistical testing: (**D**) Paired Student t-test; (**E**,**F**,**H**,**I**): Wilcoxon matched-pairs signed ranked test. Abbreviations: mLN, mesenteric lymph nodes; n.s., not significant. (**D**) Data shown as mean ± SEM, (**E**,**F**,**H**,**I**) Data shown as median ± IQR.

**Figure 2:**
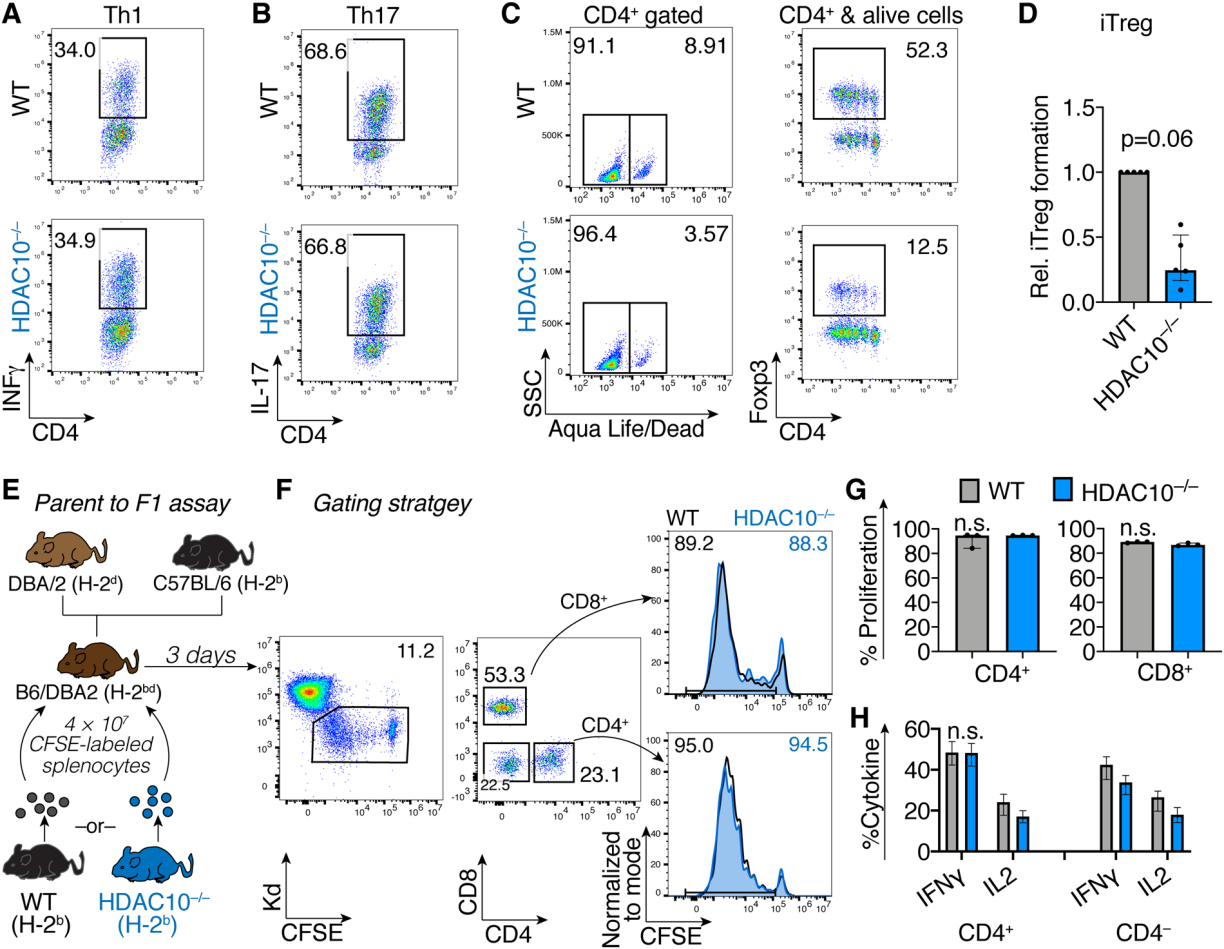
HDAC10 deletion does not impair conventional T cell function. (**A**–**C**) WT and HDAC10^−/−^ conventional T cells were co-stimulated and cultured under polarizing conditions to form Th1 (**A**), Th17 (**B**), and induced Treg (**C**,**D**). HDAC10^−/−^ Tconv showed a trend to form less Foxp3^+^ induced Treg, but significance was missed (Wilcoxon matched-pairs signed rank test). Data representative of two (**A**,**B**) and five (**C**,**D**) independent experiments. (**E**–**H**) Parent-to-F1 assay. (**E**) schematic: 4 × 10^7^ C57BL/6 (H-2^b^) WT or HDAC10^−/−^ splenocytes were CFSE-labeled, and adoptively transferred (i.v.) into C57BL/6-DBA/2 (H-2^bd^) recipients. After three days, the adoptively transferred cells were identified by their absence of H-2^d^ MHC. CD8^+^ and CD4^+^ T cells lacking HDAC10 proliferated equally well compared to WT *in vivo*. (**F**) Gating strategy. (**G**,**H**) Pooled data of CD4^+^ and CD8^+^ T cell proliferation (**G**) and cytokine production after PMA/ionomycin activation (**H**). Data pooled from three independent experiments (Mann-Whitney test). Abbreviations: n.s., not significant. Data shown as median ± IQR.

Mice lacking HDAC10 (Fig. 1A) were born at the expected Mendelian ratios and had no signs of disease or abnormal development up to one year of observation. HDAC10^−/−^ lymphatic tissues had similar CD4^+^ and CD8^+^, as well as CD44^hi^CD62L^lo^ effector/memory cells (Fig. 1B–F), as well as CD25^+^ and Foxp3^+^ T cell populations (Fig. 1G–I) compared to wild type (WT) mice. Other immune cell populations, such as B-cells (B220, CD19), natural killer cells (CD49b), and macrophages were comparable (data not shown). Next, we compared HDAC10^−/−^ and WT CD4^+^ T cell subset polarization. We observed very similar Th1 and Th17 formation (Fig. 2A,B). HDAC10^−/−^ CD4^+^CD25^−^ T cells showed a trend towards reduced Foxp3^+^ iTreg polarization upon CD3ε/CD28 mAb co-stimulation in the presence of TGF-β and IL-2 (Fig. 2C,D). To evaluate Tconv and CD8^+^ T cell function *in vivo*, we adoptively transferred CFSE-labeled WT and HDAC10^−/−^ splenocytes of C57BL/6 origin (H-2^b^) into C57BL/6-DBA2 (H-2^bd^) mice (Parent-to-F1 assay, Fig. 2E). After three days, we obtained splenocytes of the recipients and identified the adoptively transferred cells through the absence of the H-2^d^ alloantigen (Fig. 2F). We observed, that both WT and HDAC10-deficient CD4^+^ and CD8^+^ T cells proliferated equally well and produced IL-2 and IFN-γ *in vivo* (Fig. 2F–H). In summary, deletion of HDAC10 produced viable mice without apparent illness and with functional CD4^+^ and CD8^+^ T cells.

### HDAC10 deletion improves Treg function

**Figure 3:**
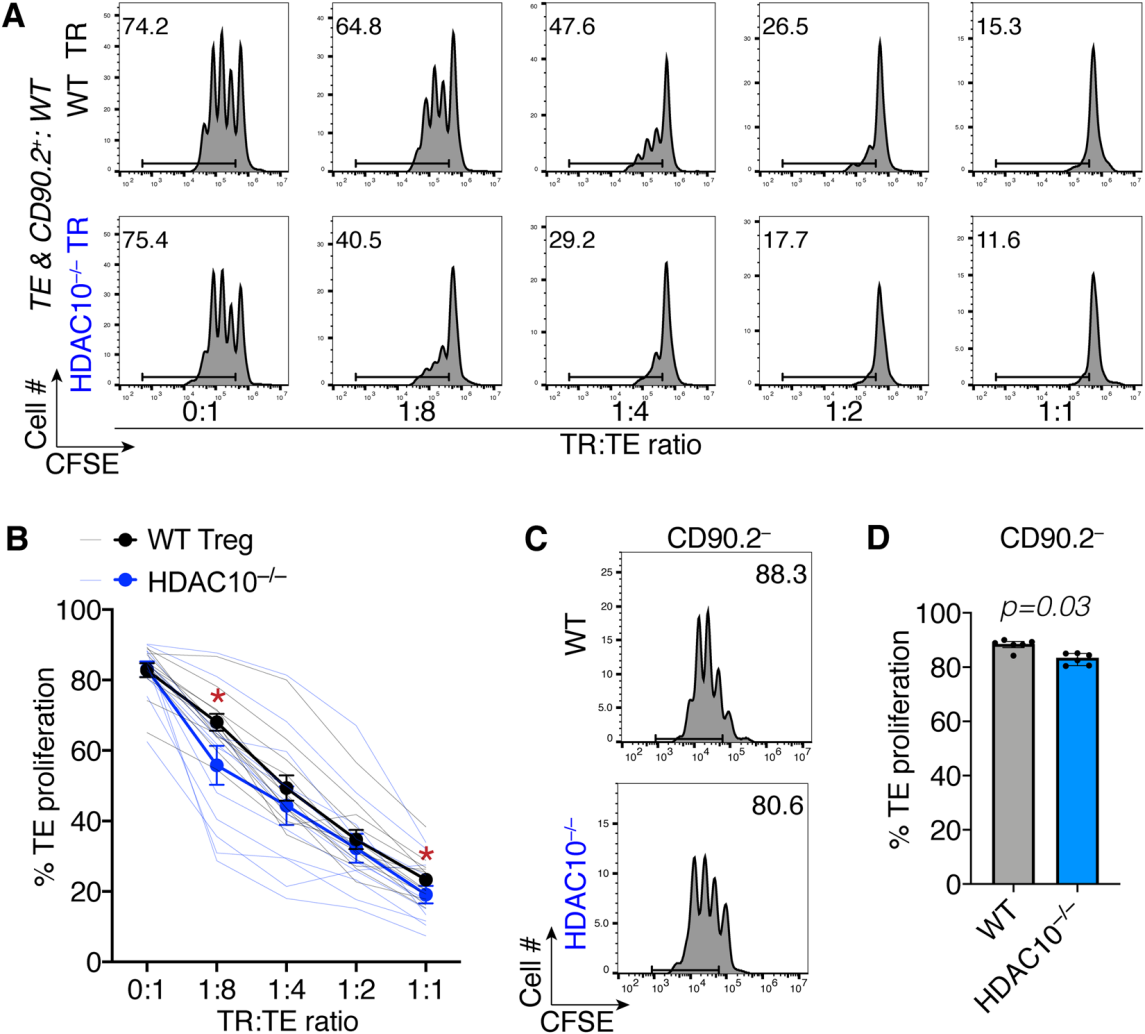
HDAC10^−/−^ Tregs have increased suppressive function. CD4+ CD25^−^ T cells were CFSE-labeled and co-stimulated with anti-CD3 mAb and WT irradiated CD90.2^−^ antigen presenting cells. Regulatory T cells (TR) were added to the stimulated T-effector (TE) cells at the indicated TR:TE ratio. After three days, proliferation of the co-stimulated T-effector cells was assessed via flow cytometry by measuring CFSE dye dilution. We combined TE, CD90.2^−^ and TR from WT or HDAC10^−/−^ mice. (**A**) Representative comparison of WT versus HDAC10^−/−^ TR suppressive fuction. (**B**) Quantitative TR suppressive function data pooled from 12 WT versus HDAC10^−/−^ TR pairings from 8 independent experiments experiments (paired Student t-test, * indicates p < 0.05). (**C**) Representative and (**B**) quantitative comparison of six WT versus HDAC10^−/−^ CD90.2^−^ pairings from two independent experiments (Wilcoxon matched-pairs signed rank test). (**B**) Data shown as mean ± SEM. (**D**) Data shown as median ± IQR.

In previous studies of the other HDAC class IIb isoform, HDAC6, we showed that HDAC6 deletion or its pharmacologic inhibition promoted Foxp3 acetylation and increased Treg suppressive function *in vitro* and *in vivo*^8,10^. These findings led us to question if HDAC10^−/−^ Treg might exhibit a similar phenotype? Indeed, compared to WT Tregs, HDAC10^−/−^ Treg showed stronger suppressive function against effector T cells proliferating *in vitro* (Fig. 3A,B), mirroring the phenotype of HDAC6-deficient Tregs. This observation led us to consider how targeting of HDAC10 might enhance the Treg suppression, including whether increased Foxp3 acetylation was involved, as with certain other HDAC phenotypes, including HDAC6, HDAC9, and Sirtuin-1^6,10^. Of note, in addition to the increased HDAC10^−/−^ Treg function, we also observed, that if the cells used to co-stimulate effector T cells in the Treg suppressive function assays (an irradiated mixed splenocyte fraction from which CD90.2^+^ T cell have been removed) originated from HDAC10^−/−^ rather than WT mice, that effector T cell proliferation was higher (Fig. 3C,D).

### HDAC10 co-precipitates with Foxp3

**Figure 4:**
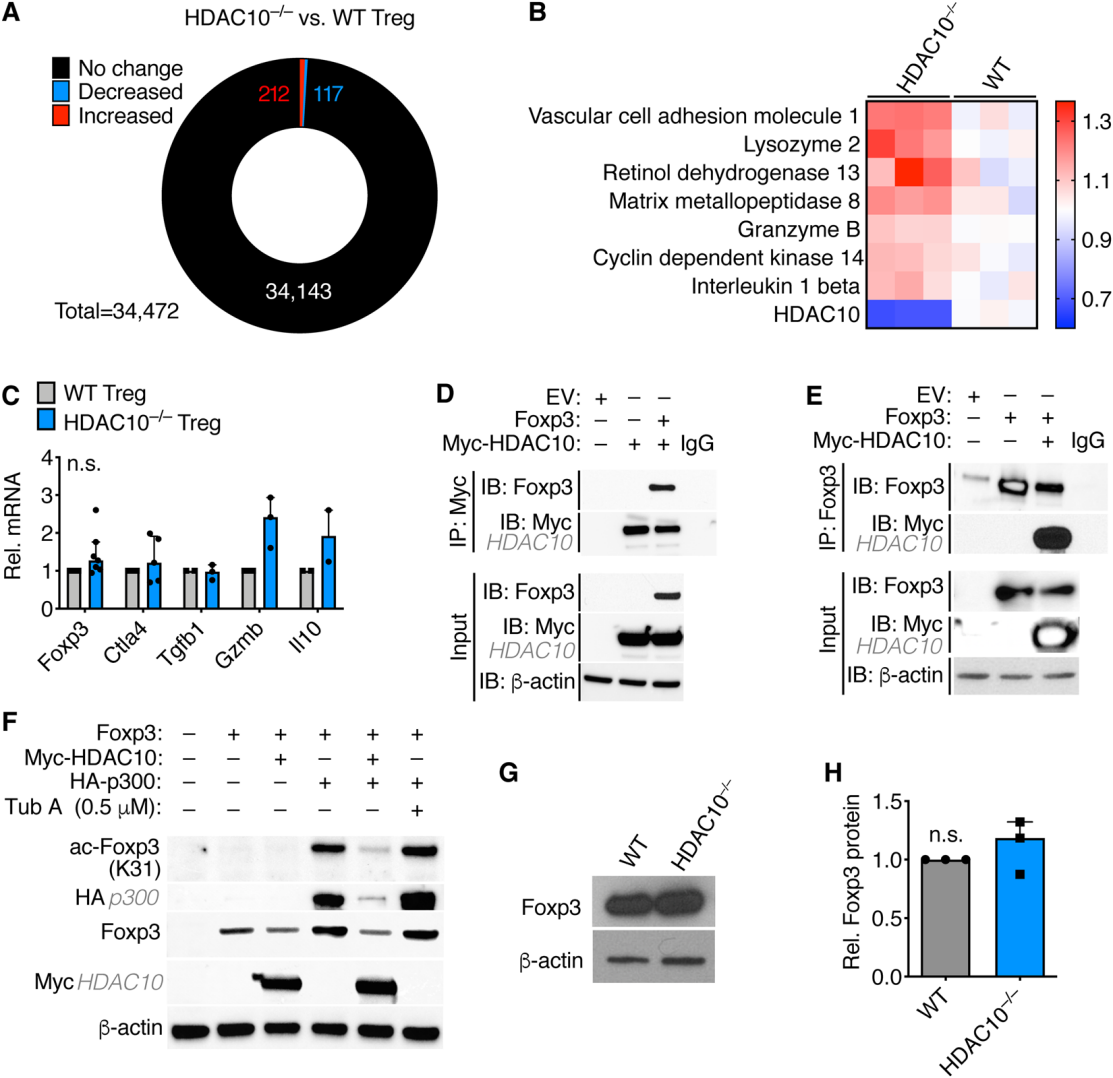
HDAC10 co-precipitates with Foxp3. (**A**, **B**) Whole-mouse-genome oligoarrays (GeneChip™ Mouse Gene 2.0 ST) of WT and HDAC10^−/−^ Treg. (**A**) Overview of differential gene expression, with 212 (0.62%) increased and 117 (0.34%) decreased genes in HDAC10^−/−^ compared to WT Treg, respectively (Student t-test, FDR < 0.1, 1.5-fold differences). (**B**) Selected differentially expressed genes of potential interest, with red indicating increased, and blue decreased gene expression in HDAC10^−/−^ Treg relative WT Treg control. Data normalized to the average of WT Treg per row. (**C**) Quantitative PCR of WT and HDAC10^−/−^ Treg confirms the granzyme B increase noted in the microarray studies (n = 2–6/group, Wilcoxon matched-pairs signed rank test). (**D**–**F**) Evaluation of Foxp3 and HDAC10 interaction through overexpression. 293 T cells were transfected with empty vector, Foxp3, myc-tagged HDAC10, and/or HA-tagged p300, and proteins were extracted, immunoprecipitated (IP) and immunoblotted (IB) as indicated. HDAC10 and Foxp3 are shown to bind via HDAC10 (**D**) and Foxp3 (**E**) pulldown. (**F**) Overexpression of HDAC10 diminishes Foxp3 K31 acetylation and p300 expression. Tubastatin A (Tub A) is a class IIb HDAC inhibitor targeting both HDAC6 and HDAC10^29^. (**G**,**H**) Immunoblot showing Foxp3 protein expression in HDAC10^−/−^ and WT Treg. (**G**) representative and (**H**) quantitative data pooled from three independent experiments (Student t-test). (**C**,**H**) Data shown as median ± IQR.

To detect differences in gene expression between WT and HDAC10^−/−^ Treg, we isolated RNA from naïve CD4^+^CD25^+^ Tregs and conducted whole-mouse-genome oligoarrays studies (GeneChip™ Mouse Gene 2.0 ST, Thermo Fisher Scientific). The effect of HDAC10 deletion on Treg gene expression was limited. Under non-stringent statistical criteria (Student t-test FDR <0.1, 1.5-fold differential expression), <1% of genes were differentially expressed (Fig. 4A, Supplementary Excel File). We noted that *Granzyme-b* mRNA was increased in HDAC10^−/−^ Treg (Fig. 4B), which was confirmed by qPCR (Fig. 4C). Foxp3 mRNA showed a trend to higher expression but missed statistical significance, as did other Treg-associated genes such as *Ctla4*, *Tgfb1* and *Il10*. (Fig. 4C). Next, we examined if HDAC10 and Foxp3 interacted. We transfected 293 T cells with Foxp3 and HDAC10, and observed binding, regardless of whether the complex was immunoprecipitated by HDAC10 and blotted by Foxp3, or vice versa (Fig. 4D,E). The immunoprecipitation between Foxp3 and HDAC10 led us to hypothesize that HDAC10 may deacetylate Foxp3. We observed that Foxp3-K31 acetylation, mediated by co-transfection of 293 T cells with Foxp3 and the histone/protein acetyl transferase p300^23^, was decreased with HDAC10 co-transfection (Fig. 4F). However, we could not discern if this was the result of HDAC10 deacetylating Foxp3 or destabilizing p300 expression. 293 T cell overexpression systems are helpful for examining biochemical interactions but have limitations. Increased Foxp3 acetylation has been observed to lead to better Foxp3 preservation (higher total Foxp3) and DNA binding by us and other groups^3,4,24^. To evaluate if loss of HDAC10 leads to more Foxp3, we conducted Western blotting. We did not observe a significant change in total Foxp3 protein expression (Fig. 4G,H). Taken together, Foxp3 and HDAC10 expressed in 293 T cells were found to co-precipitate, but we could not confirm an increase in Foxp3 protein acetylation, and it remains unclear if this step is mechanistically important to the increased HDAC10^−/−^ suppressive function.

### HDAC10 deletion has no effects on Treg metabolism

**Figure 5:**
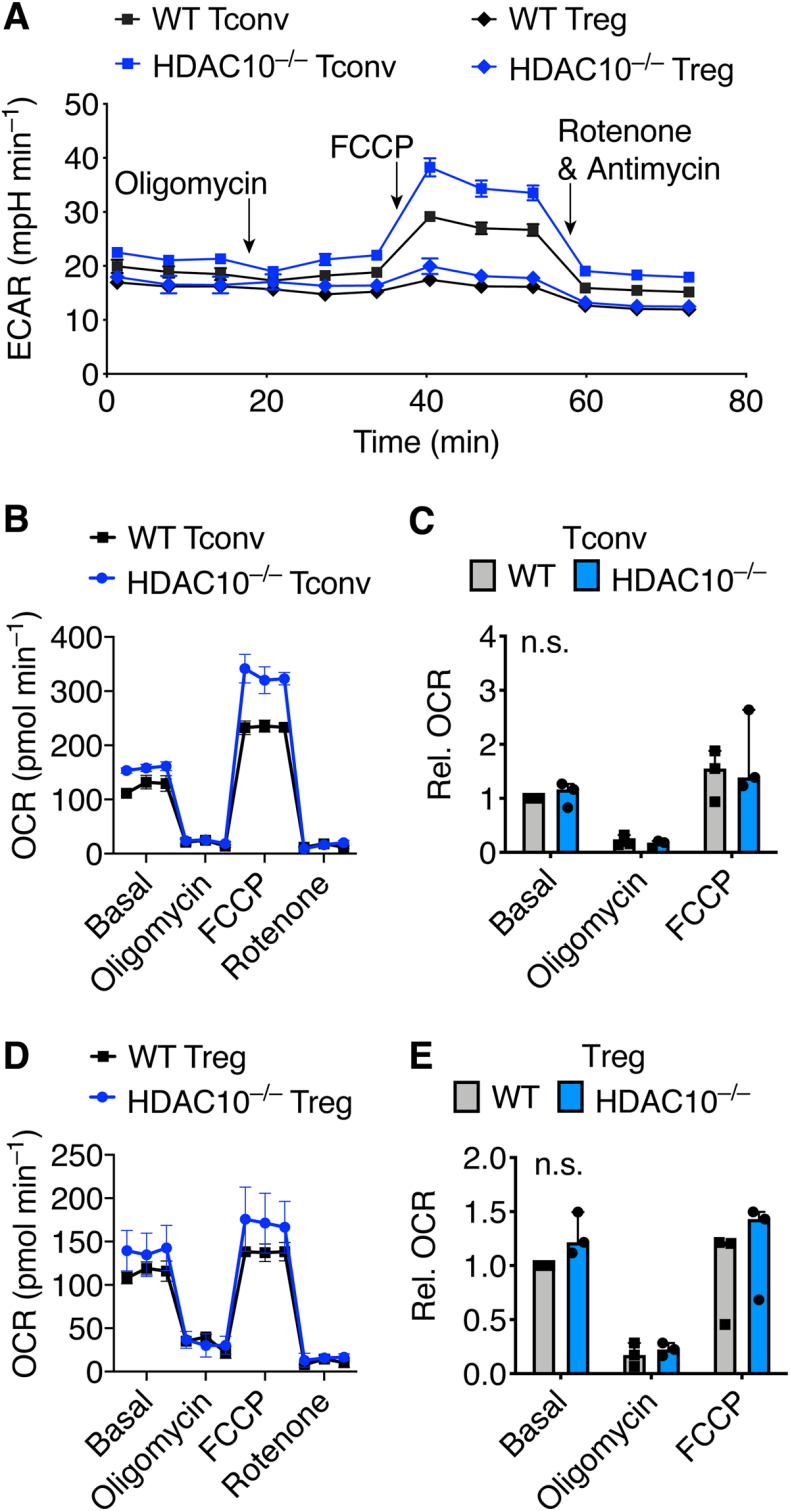
Bioenergetic profile of HDAC10 deficient conventional and regulatory T cells. Bioenergetic measurements evaluating HDAC10^−/−^ and wild type (WT) Tconv and Treg, assessing extracellular acidification (ECAR, **A**), as well as oxygen consumption (OCR, **B**–**E**) using Seahorse. (**A**) Tconv and Treg were CD3ε/CD28 mAb co-stimulated for 16 hours. Treg exhibited, as expected, lower ECAR than Tconv, but no difference between HDAC10^−/−^ and WT was noted. (**B**–**E**) Tconv (**B**,**C**) and Treg (**D**,**E**) co-stimulated for 2 hours. (**A**,**B,D**) Representative and (**C**,**E**) pooled data from three independent experiments normalized to WT basal respiration (Wilcoxon matched-pairs signed rank test). Abbreviation: n.s., not significant. (**A,B,D**) Data shown as mean ± SEM. (**C,E**) Data shown as median ± IQR.

HDAC10 has been reported to be highly expressed in lung cancer tissues where it is associated with a poor prognosis^25^. HDAC10 also promoted AKT phosphorylation in lung cancer cell lines^21^, which could affect metabolism by switching to a glycolytic phenotype^26^, and potentially have detrimental effects on Treg function^27^. We evaluated HDAC10^−/−^ Tconv and Treg bioenergetic function with and without CD3/CD28 co-stimulation. We did not find a difference in basal glycolytic activity (Fig. 5A) or in Tconv oxygen consumption (Fig. 5B,C). We noticed a trend in HDAC10^−/−^ towards an increased uncoupled oxygen consumption rate (Fig. 5D,E), not unlike that seen in HDAC6 deficient Tregs^28^. Overall, the differences are small and unlikely to be a major contributor to HDAC10^−/−^ Treg function.

### Tregs lacking HDAC10 alleviates autoimmune colitis

**Figure 6:**
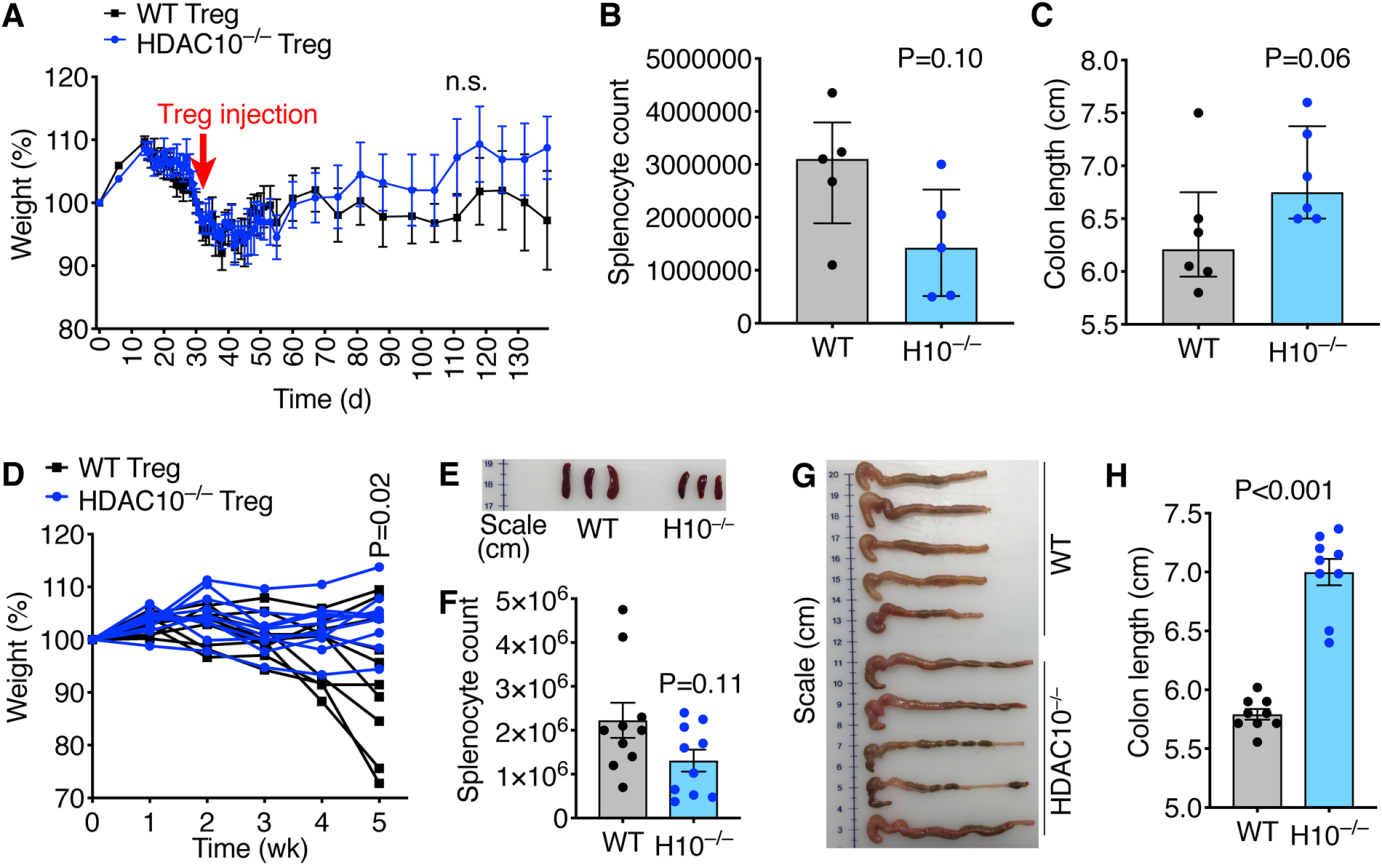
HDAC10^−/−^ Treg cells improve autoimmune colitis outcomes. (**A**–**C**) Autoimmune colitis rescue model: B6/Rag1^−/−^ mice were adoptively transferred with 1 × 10^6^ wild type (WT) CD4^+^CD25^−^ Tconv i.p. (day 0), and were closely monitored for weight loss. On day 33 after adoptive transfer of the Tconv, the mice received 5 × 10^5^ CD4^+^CD25^+^ Treg from either WT or HDAC10^−/−^ (H10^−/−^) mice i.p. (**A**) Weight tracking of B6/Rag1^−/−^ mice receiving Treg from either WT or HDAC10^−/−^ donors. (**B**,**C**) At the end of the experiment (day 139), HDAC10^−/−^ Treg recipients showed a trend to less splenocyte counts (**B**) and longer colon length (**C**), however, significance was missed (Mann Whitney test, 5–6 mice per group). (**D**–**H**) Autoimmune colitis prevention model: B6/Rag1^−/−^ mice were adoptively transferred i.p. with 1 × 10^6^ wild type (WT) CD4^+^CD25^−^ Tconv together with 5 × 10^5^ CD4^+^CD25^+^ Treg from either WT or HDAC10^−/−^ donors. (**D**) B6/Rag1^−/−^ mice receiving HDAC10^−/−^ Treg developed less weight loss (unpaired Student t-test, 10/group). (**E**,**F**) HDAC10^−/−^ Treg recipients exhibited less smaller spleens. (**E**) Shows representative photograph, (**F**) splenocyte counts (unpaired Student t-test). (**G**,**H**) HDAC10^−/−^ Treg recipients were found to have longer colons. (**G)** Representative photograph, (**H**) colon measurements (unpaired Student t-test). Data shown as mean ± SEM (**A**,**F**,**H**), median ± IQR (**B**,**C**), and individual replicates (**D**).

**Figure 7:**
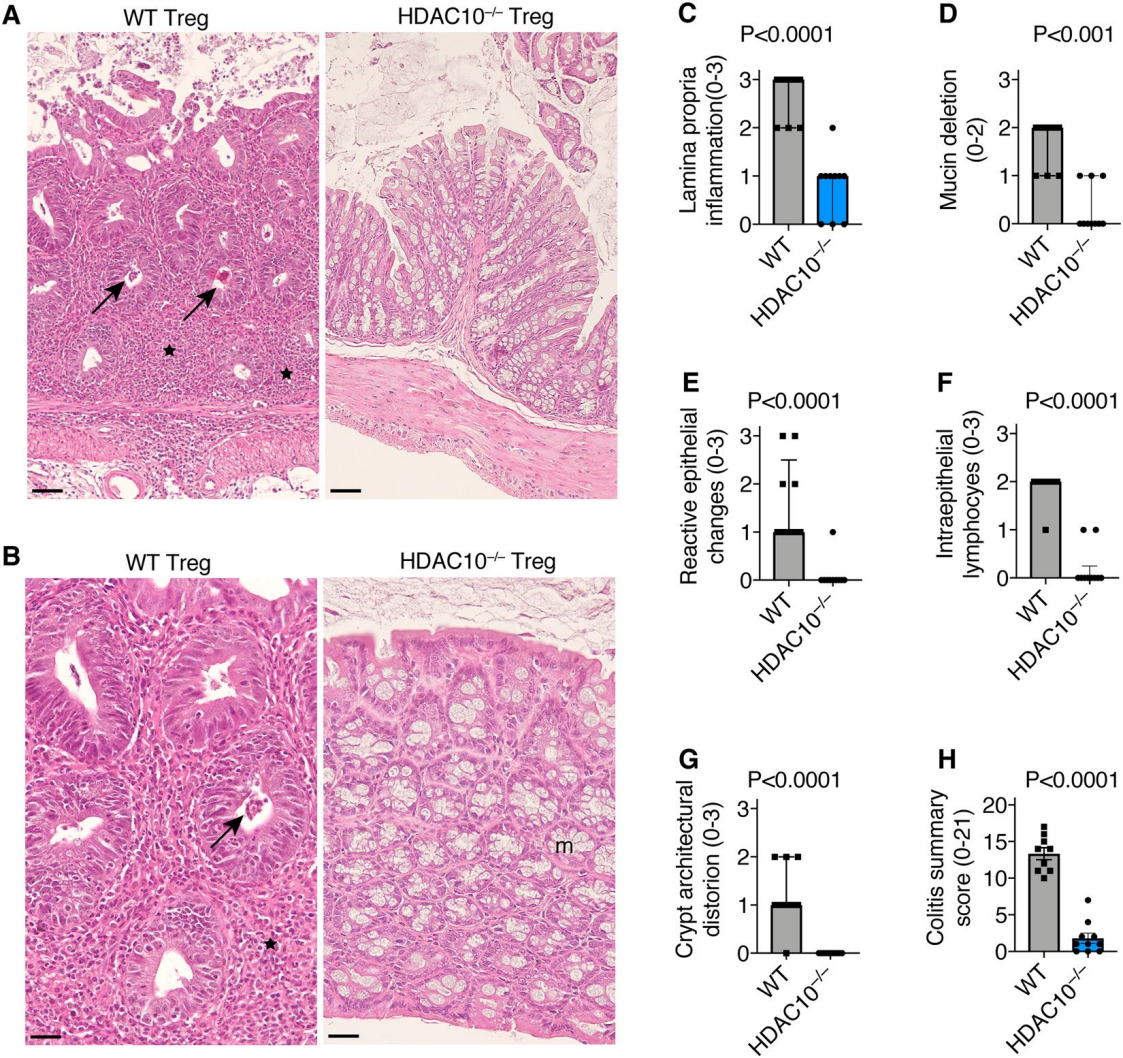
HDAC10^−/−^ Treg cells limit autoimmune colitis. Histologic analysis of B6/Rag1^−/−^ effector and regulatory T cells from the prevention colitis model (Fig. 6D). (**A**,**B**) Representative histology (H&E) with low (scale bar: 125 μm) and high magnifications (scale bar: 250 μm). (**A**) Mice receiving WT Tconv and WT Tregs developed severe colitis, with transmural inflammation, ulceration, depletion of goblet cells, disruption of crypt architecture and reactive epithelial changes, whereas mice receiving WT Tconv and HDAC10^−/−^ Tregs typically lacked these events and appeared largely normal. (B) Higher magnifications showing crypt abscesses (arrow), dense leucocytic infiltration (★), loss of mucinous glands and loss of crypt architecture in mice receiving WT Tregs compared to normal histology in those receiving HDAC10^−/−^ Treg cells, where normal mucinous glands are indicated by (m). (**C**–**H**) Pooled data from blinded histological analysis. (**C**–**G**) Data shown as median ± interquartile range, p-values represent Mann-Whitney test. (**H**) Data shown as mean ± SEM, p-value represents Student t-test.

We questioned if the increased HDAC10^−/−^ Treg suppressive function observed in our *in vitro* studies translated into *in vivo* models of autoimmune disease and transplantation. We evaluated two autoimmune colitis models, rescue and prevention. In the colitis rescue model, B6/Rag1^−/−^ mice were adoptively transferred i.p. with 10^6^ WT Tconv, and then observed for weight loss and clinically signs of colitis. By day 33, the mice had developed colitis and weight loss, and were randomly assigned to receive 5 × 10^5^ Treg i.p. from either WT or HDAC10^−/−^ donor mice for colitis rescue. B6/Rag1^−/−^ mice receiving HDAC10^−/−^ Treg showed a trend to improved weight outcomes (Fig. 6A), reduced splenocyte counts and preservation of colon length (Fig. 6B,C), however, the differences were not statistically significant. In the colitis prevention model, B6/Rag1^−/−^ mice were adoptively transferred i.p. with 1 × 10^6^ WT CD4^+^CD25^−^ Tconv together with 5 × 10^5^ CD4^+^CD25^+^ Treg from either WT or HDAC10^−/−^ donors simultaneously. Here, the differences were stronger. B6/Rag1^−/−^ mice receiving HDAC10^−/−^ Treg showed less weight loss (Fig. 6D), smaller spleens and less splenocytes (Fig. 6E,F), as well as less colon shortening and thickening (Fig. 5G,H). This was matched by less inflammation upon histologic examination, whereby colon samples from B6/Rag1^−/−^ mice receiving WT, but not HDAC10^−/−^ Treg showed more prominent colitis including transmural inflammation, ulceration, reactive epithelial changes and crypt abscesses (Fig. 7A,B). These observations were quantified by blinded analysis (Fig. 7C–H). In conclusion, our data show that HDAC10^−/−^ Treg can alleviate autoimmune colitis.

### HDAC10^−/−^ mice show prolonged MHC-mismatched cardiac allograft survival

**Figure 8:**
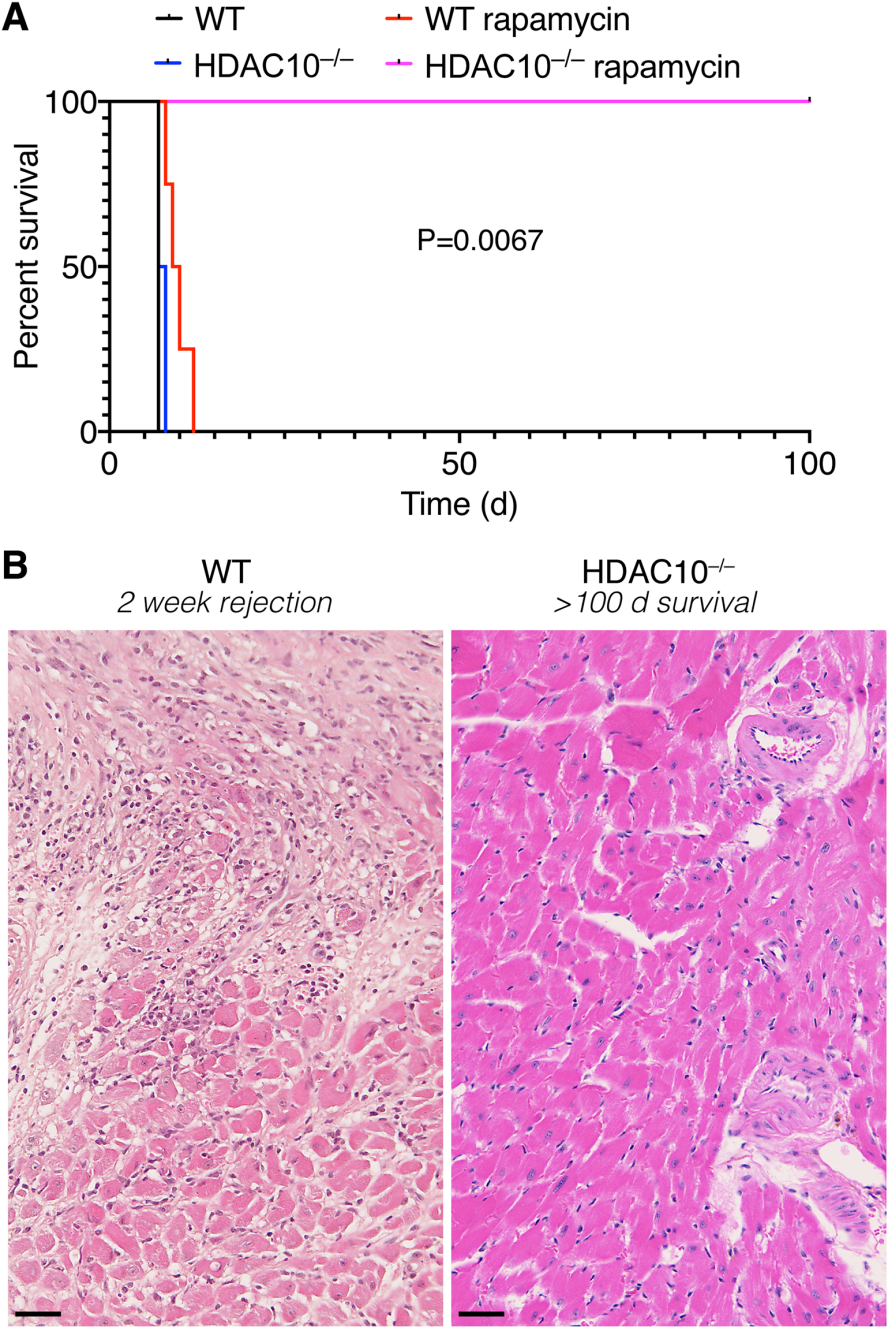
Loss of HDAC10, in conjunction with low dose rapamycin, prolongs cardiac allograft survival. (**A**) Cardiac allograft survival of BALB/c hearts (H-2^d^) transplanted into C57BL/6 (H-2^b^) recipients. Recipients receiving low dose rapamycin (0.1 mg kg^−1^ d^−1^ i.p. for 14 days post-transplant) and lacking HDAC10 showed >100 days of cardiac allograft survival. Survival curve data shown from four mice per group. P-value indicates Log-rank (Mantel-Cox) test comparing rapamycin receiving wild type (WT) and HDAC10^−/−^ recipients. Data pooled from four mice per group. (**B**) Representative H&E histology of BALB/c cardiac allografts recovered from WT mice (left) receiving 0.1 mg kg^−1^ d^−1^ i.p. for 14 days post-transplant at 2 weeks, and from corresponding HDAC10^−/−^ recipients harvested after 100 days post-transplant. Hearts from WT recipients end-stage acute rejection with mononuclear cell infiltrates and myocyte necrosis, whereas those from HDAC10^−/−^ recipients showed sparse mononuclear cell infiltrates, preservation of myocardial histology and an absence of transplant arteriosclerosis or interstitial fibrosis. Scale bar: 250 μm.

Mice lacking HDAC6, and mice treated with an HDAC6 inhibitor, show prolonged renal allograft survival^8^. We questioned if loss of HDAC10 would produce a similar phenotype? We transplanted MHC-mismatched BALB/c cardiac allografts into WT and HDAC10^−/−^ recipient mice (H-2^d^ to H-2^b^), and monitored allograft survival daily by palpation of ventricular contractions. In the absence of further treatment, both WT and HDAC10^−/−^ recipients rejected their cardiac allografts rapidly (Fig. 8A). However, addition of subtherapeutic injections of rapamycin (0.1 mg kg^−1^ d^−1^ i.p. for 14 days post-transplant) elicited a strong difference between WT and HDAC10^−/−^ recipients, where the latter did not reject their cardiac allografts for >100 days post-transplant (Fig. 8A). The cardiac allografts recovered from HDAC10^−/−^ did not only maintain their function >100 days (p < 0.01), but, unlike WT mice at 2 weeks post-transplant, also showed no signs of rejection and maintained normal cardiac histologic architecture (Fig. 8B). In conclusion, in contrast to WT recipients, HDAC10^−/−^ mice receiving a brief course of low-dose rapamycin maintained their fully MHC-mismatched cardiac allografts indefinitely.

## Discussion

Our studies of the HDAC10 immune phenotype reveal an overall immunosuppressive effect with HDAC10 deletion. We observed that Foxp3^+^ Treg cells lacking HDAC10 had increased suppressive function *in vitro*, and HDAC10^−/−^ mice accepted MHC-mismatched cardiac allografts long-term when treated with 14 days of low-dose rapamycin, while B6/Rag1^−/−^ receiving HDAC10^−/−^ Treg showed increased resistance to development of autoimmune colitis *in vivo*. However, it is important to also consider additional subsets of immune cells that may be altered through HDAC10 deletion. Our *in vitro* studies had indicated that irradiated CD90.2^−^ splenocytes, including antigen presenting cells that provide co-stimulatory signals in the Treg suppression assays, showed decreased stimulatory effects on effector T cells when lacking HDAC10. This finding is consistent with prior reports on HDAC10 deletion on antigen-presenting cells^22^. It is possible that decreased co-stimulatory signals in HDAC10^−/−^ transplant recipients could contribute to the observed prolonged allograft survival. Furthermore, differential effects of rapamycin on HDAC10-deficient versus wild type mice need to be considered. That said, Treg are likely to play a somewhat important role, considering that the origin of Tregs (HDAC10^−/−^ vs WT) was the only variable in the colitis prevention model.

Our data are congruent with the effects of genetic or pharmacologic HDAC6 targeting, as well global pharmacologic class IIb HDAC inhibition, which is reassuring for the further development of HDAC isoform-selective immunosuppressive therapeutics. Indeed, two recent reports indicate that development of HDAC10-selective inhibitors is feasible based upon targeting of the interaction of a hydrogen-bond between a cap group nitrogen and the gatekeeper residue Glu272 that is responsible for potent HDAC10 binding^29,30^. The effects of such inhibitors on immune functions will need to be carefully characterized once they become available, since the screening assays used for their development involved FRET-based displacement of HDAC10-bound ligands rather than enzymatic inhibition. Such inhibitors may potentially act on the lysine deacetylase activity of HDAC10 at least in some contexts, whereas in others, effects on the recently identified polyamine deacetylase activity of HDAC10 may be dominant^31^.

Beyond targeting class IIb HDACs for therapeutic immunosuppression, our data also indicate a note of caution when considering HDAC10 as a therapeutic target in cancer, which has been suggested by several groups. Increased HDAC10 expression has been associated with several malignancies, including lung and gastric cancer and neuroblastoma, and was been linked with poor outcomes^17,25,32–34^. A number of mechanisms have been proposed. E.g. Yang *et al*. reported that HDAC10 promotes AKT phosphorylation in lung cancer cells^21^, and AKT phosphorylation is known to propagate cell growth, proliferation and survival^35^. However, if HDAC10 inhibition improves Treg immunosuppressive capacity, this could have counterproductive effects, given the importance of Tregs in cancer pathology^36^. Beyond immunosuppressive concerns for anti-tumor HDAC10 targeting, HDAC10 has also been reported to impair matrix metalloproteinase-2 and −9 expression^37^, which were important in metastasis formation^38^.

In conclusion, HDAC10 deletion leads to stronger Foxp3^+^ Treg cells with increased suppressive function *in vivo*. Pharmacologic targeting of HDAC10 and/or class IIb HDACs in general may be a promising strategy to augment immunosuppressive therapy.

## Methods

### Mice

We purchased B6/Rag1^−/−^ and C57BL/6 (both H-2^b^) mice of either wild-type (WT) or HDAC10^−/−^ (B6N(Cg)-*Hdac10*^*tm1.1(KOMP)Mbp*^/J) origin^39^, as well as BALB/c (H-2^d^) and B6/DBA2 (H-2^bd^) from he Jackson Laboratory (Bar Harbor, ME). Mice were housed under specific-pathogen-free conditions and studied using protocols approved by the Institutional Animal Care and Use Committees of the Children’s Hospital of Philadelphia (16–000561 and 17-000746). All animals reproduced at expected Mendelian ratios and were housed under standard conditions (group housing up to five per cage), except B6/Rag1^−/−^ which require high barrier housing (also group housing up to five per cage).

### Cell lines

293 T (CRL-3216™) were obtained from American Type Culture Collection (ATCC), suggested to be female in origin.

### Cell culture media and cell culture conditions

For standard cell culture medium, we used RPMI 1640 medium supplemented with 10% fetal bovine serum (FBS), penicillin (100 U × mL^−1^), streptomycin (100 µg × mL^−1^), and 55 nM β_2_-mercaptoethanol. All bioenergetic measurements used Agilent Seahorse XF base medium (cat. #102353-100) from Aligent Technologies (Santa Clara, CA) plus 10 mM D-glucose, 1 mM Na pyruvate, and 2 mM L-glutamine.

### Antibodies, plasmids and reagents

We purchased CD4 (BD Bioscience, Pacific blue, clone RM4-5, #558107), CD8 116 (PE-Cy7, eBioscience, clone 53-6.7, cat. #25-0081), Foxp3 (PE-Cy5, eBioscience, clone FJK-16s, cat. #15-5773), CD3 (clone 145-2C11, cat. #553057), CD28 (clone 37.51, cat. #553294) mAbs from BD Bioscience, and IFN-γ (Alexa Fluor® 647, cat. #557735) and IL-17Α (Alexa Fluor® 647, cat. #560184) from BD Pharmingen. For immunoblotting, we used HDAC10 (Abcam, cat. #108934), β-actin (Cell Signaling, cat. #5125), MYC-tag (Cell Signaling, cat. #2276 S), HA-tag (Cell Signaling, cat. #3724 S) and Foxp3 (eBioscience, cat. #14-5777-82) antibodies. For cell culture, we used RPMI-1640 medium supplemented with 10% fetal bovine serum (FBS), penicillin (100 U mL^−1^), streptomycin (100 µg mL^−1^), and 55 nM β_2_-mercaptoethanol. HDAC10 plasmid was purchased from Origene (cat. #MR209891), p300 plasmid was provided by Dr. Xiao-Jiao Yang (McGill University, Montreal, Canada), and the Foxp3 MinR1 plasmid was previously described^4^.

### Bioenergetic measurements

We measured bioenergetic functions using XF24 and XF96 analyzers (Seahorse Biosciences, North Billerica, MA). Plates were coated using Cell-Tak (BD Biosciences)^11^. Isolated T cells were plated in unbuffered XF Assay Media, and then incubated for 30–60 min at 37 °C without CO_2_. For our T cell studies, we used 1 × 10^6^ cells per well for XF24, and 2 × 10^5^ cells per well for XF96 assays. To enhance cell adherence, plates were spun at room temperature for 5 min at 400 g. Three baseline measurements of OCR and ECAR were taken and cells were then exposed sequentially to oligomycin A, cyanide-4-[trifluoromethoxy]phenylhydrazone (FCCP) and rotenone and/or antimycin A. We used 1.25 µM oligomycin, 0.5 µM FCCP and either 1 µM rotenone followed by 1.8 µM antimycin A in XF24, or 1 µM rotenone & antimycin A combined in XF96 experiments. Three readings were taken after each sequential injection. Instrumental background was measured in separate control wells using the same conditions without biological material. Seahorse reagents were purchased from Agilent Technologies (Wilmington, DE). Data were analyzed using Wave (Agilent), Excel and Prism.

### Cardiac allografting

We transplanted BALB/c hearts (H-2^d^) into C57BL/6 recipients (H-2^b^); donor and recipient mice were female and aged 8–12 weeks. We chose female mice to minimize fighting injuries and age 8–12 weeks to enable sufficiently large blood vessels to enable microsurgeries. Some recipients received low-dose rapamycin (0.1 mg kg^−1^ day^−1^) for two weeks, as indicated. Allograft survival was assessed by palpation, and confirmed by histology after 100 days.

### Cell isolation and flow cytometry

Spleen and peripheral lymph nodes were harvested and processed to single cell suspensions of lymphocytes. Red blood cells were removed with hypotonic lysis. We used magnetic beads (Miltenyi Biotec, San Diego, CA) for isolation of Tconv (CD4^+^CD25^−^), Treg (CD4^+^CD25^+^), and antigen presenting cells (CD90.2^−^). Cells of interest were analyzed flow cytometry. All flow cytometry data was captured using Cyan (Dako) as well as Cytoflex (Beckman Coulter, Brea, CA) and analyzed using the FlowJo 10.2 software. We used phosphate buffered saline with 2% FBS as flow buffer in all experiments.

### Colitis

We tested the ability of Tregs to prevent the development of colitis, and to rescue mice with established colitis. In the prevention model, B6/Rag1^−/−^ mice were adoptively transferred i.p. with 1 × 10^6^ CD4^+^CD25^−^ Tconv cells from WT mice, and simultaneously received 5 × 10^5^ CD4^+^CD25^+^ Treg i.p. from WT or HDAC10^−/−^ mice. Mice were monitored for weight, gross blood in their stool, and other clinical parameters as previously reported^7^. In the rescue model 9–10-week-old female B6/Rag1^−/−^ mice were adoptively transferred i.p. with 1 × 10^6^ CD4^+^CD25^−^ Tconv cells from WT mice. After clinically significant colitis developed (with 5–15% of weight loss), the B6/Rag1^−/−^ mice were randomized to treatment groups, receiving of 5 × 10^5^ CD4^+^CD25^+^ Treg, i.p., from either WT or HDAC10^−/−^ donor mice. The mice were then monitored as noted for the prevention model.

### Histology

Cardiac allografts and colon samples were fixed in 10% neutral buffered formalin, routinely processed and embedded in paraffin. Histologic sections for light microscopy were cut to a thickness of 4 µm and stained with hematoxylin and eosin (H&E) and were reviewed by a pathologist (W.W.H.) blinded to treatment conditions. Histologic findings were characterized using a previously reported scoring system^40^: (1) degree of lamina propria inflammation graded 0–3; (2) degree of mucin depletion as evidenced by loss of goblet cells graded 0–2; (3) reactive epithelial changes (nuclear hyperchromatism, random nuclear atypia, increased mitotic activity) graded 0–3; (4) number of intraepithelial lymphocytes per high power field within crypts graded 0–3; (5) degree of crypt architectural distortion graded 0–3; (6) degree of inflammatory activity (infiltration of neutrophils within lamina propria and crypt epithelium, “cryptitis”) graded 0–2; (7) degree of transmural inflammation graded 0–2; and (8) degree of mucosal surface erosion up to total surface ulceration graded 0–2. The total histopathologic score (0–21) was determined from the sum of the scores for each parameter to reflect the overall degree of inflammation within each specimen.

### Immunoblotting

Immunoblotting was performed as previously reported^10^. Purified cells of interested were lysed in radioimmunoprecipitation assay (RIPA) buffer with Halt protease inhibitor (Thermo Fisher Scientific). Protein concentration was determined by photometry (iMark™ Microplate Absorbance Reader, Bio-Rad) using bovine serum albumin as standard (Cat. #500–0207, Bio-Rad Laboratories, Hercules, CA). Samples were mixed with Laemmli sample buffer containing 2-mercaptoethanol (Bio-Rad Laboratories, Hercules, CA), and loaded onto Mini-PROTEAN TGX^TM^ 4 to 15% gradient gels (Bio-Rad) and underwent electrophoretic separation. Proteins were then transferred to PolyScreen PVDF Hybridization Transfer Membranes (PerkinElmer, Waltham, MA). Membranes were cut according to the molecular weights of the proteins of interest (as indicated with Precision Plus Protein Dual Color, Bio-Rad) and incubated with primary and horseradish peroxidase (HRP)–conjugated secondary antibodies. We used Super Signal West Pico chemiluminescent substrate (Thermo Fisher Scientific) and X-OMAT Blue XB Film (Kodak, Rochester, NY) or the ChemiDoc^TM^ imaging system (Bio-Rad), using Image Lab^TM^ software (Bio-Rad) for image file export. Images were processed with Adobe Photoshop Creative Cloud (grayscale conversion and auto contrast function). Densitometric analysis was performed using ImageJ64 version 1.45 S (https://imagej.nih.gov/ij/).

### RNA extraction and quantitative polymerase chain reaction

RNA was extracted using RNeasy kits (Qiagen, Hilden, Germany), and RNA integrity and quantity were analyzed by photometry (Nanodrop 2000, Thermo Fischer). Reverse transcription and qPCR were performed as reported^9–11^. Isolated RNA was reverse transcribed to cDNA with random hexamers and amplified (PTC-200; MJ Research). Primer sequences for target genes were used for quantitative PCR amplification of total cDNA. All primers were purchased from Applied Biosystems. Differences in cDNA input were corrected by normalizing signals obtained with specific primers for 18 S rRNA. Relative quantitation of target cDNA was determined by the formula 2^−ΔCT^, with ΔCT denoting fold increases above the set control value (resting Tconv). Data was analyzed using StepOnePlus^TM^ (Applied Biosystems), Excel, and Prism.

### Microarray

WT and HDAC10^−/−^ Treg were isolated and RNA extracted as noted above. Microarray experiments were performed using whole-mouse-genome oligoarrays (GeneChip™ Mouse Gene 2.0 ST Array), and array data were analyzed using Transcriptome Analysis Console 4.0 software (ThermoFisher Scientific). Array data were subjected to robust multiarray average normalization. To assess differential gene expression, fold changes of up- and downregulated genes were calculated, and significance assessed via Student t-test. Data with a false discovery rate <0.1 and >1.5-fold differential expression were included in the analysis.

### T cell function studies

Treg suppression, cytokine production, as well as inducible Treg (iTreg) formation and Th1 and Th17 polarization were conducted as reported^13^. For Treg suppression assays, purified Tconv cells were labeled with carboxyfluorescein succinimidyl ester (CFSE, Thermo Fisher) and stimulated with irradiated antigen presenting cells plus CD3ε mAb (1 µg mL^−1^, BD Pharmingen). After 72 h, proliferation of Tconv cells was determined by flow cytometric analysis of CFSE dilution. For conversion to Foxp3^+^ Tregs, Tconv cells were incubated for 3–5 days with CD3ε/CD28 mAb beads, plus TGF-β (3 ng mL^−1^) and IL-2 (25 U mL^−1^), and analyzed by flow cytometry for Foxp3^+^ iTreg. For Th1 polarization, splenocytes were stimulated with plate-bound CD3ε mAb (2 μg mL^−1^, incubated at 37 °C for 1 hr, BD Pharmingen) and IL-12 (10 ng mL^−1^; eBioscience, cat. #14–8121–80), and anti–IL-4 (10 μg mL^−1^; BD Bioscience, cat. #554432). Cells were cultured for four days and analyzed by flow cytometry. For Th17 conversion, splenocytes were depleted of CD8^+^ T cells using Miltenyi CD8 microbeads, and cultured with soluble CD3ε and CD28 mAb (1 μg mL^−1^ each) for four days in the presence of anti-IL-4 and anti-IFN-γ mAbs (20 μg mL^−1^), TGFβ (1 ng mL^−1^), and IL-6 (10 ng mL^−1^). For intracellular IL-17 staining, cells were stimulated with 30 ng mL^−1^ phorbol 12-myristate 13-acetate (PMA) and 1 μM ionomycin (Sigma Aldrich) for 5 hours in the presence of GolgiStop reagent (BD Biosciences). We used the Fixation/Permeabilization Buffer set from eBioscience for intranuclear staining.

### Quantification and Statistical analyses

Statistical analysis was conducted using GraphPad Prism 8 software. All data were tested for normal Gaussian distribution of variables using the D’Agostino-Pearson normality test. All normally distributed data were displayed as means ± standard error of the mean (SEM) unless otherwise noted, while non-normally distributed data were displayed as median ± interquartile range (IQR). Measurements between two groups were performed with an unpaired Student’s t-test if normally distributed, or Mann-Whitney U test if otherwise. For paired samples, we used a paired Student-t test. Allograft survival was assessed using a log-rank (Mantel-Cox) test. Statistical parameters for each experiment can be found within the corresponding figure legends.

## Supplementary information

Supplementary Dataset 1.

## Data Availability

The microarray dataset for this study can be found in the in the Gene Expression Omnibus database (www.ncbi.nlm.nih.gov/geo), under the accession number GEO: GSE131794. Contact for reagent and resource sharing: Further information and requests for resources and reagents should be directed and will be fulfilled by the Lead Contact, Wayne W. Hancock (whancock@pennmedicine.upenn.edu).

## References

[1] Feuerer M, Hill JA, Mathis D, Benoist C (2009). Foxp3+ regulatory T cells: differentiation, specification, subphenotypes. Nat. Immunol..

[2] Yano Hiroshi, Andrews Lawrence P., Workman Creg J., Vignali Dario A. A. (2019). Intratumoral regulatory T cells: markers, subsets and their impact on anti‐tumor immunity. Immunology.

[3] van Loosdregt J (2010). Regulation of Treg functionality by acetylation-mediated Foxp3 protein stabilization. Blood.

[4] Liu Y, Wang L, Han R, Beier UH, Hancock WW (2012). Two lysines in the forkhead domain of foxp3 are key to T regulatory cell function. PLoS One.

[5] Wang L (2018). Histone/protein deacetylase inhibitor therapy for enhancement of Foxp3+ T-regulatory cell function posttransplantation. Am. J. Transpl..

[6] Tao R (2007). Deacetylase inhibition promotes the generation and function of regulatory T cells. Nat. Med..

[7] de Zoeten EF, Wang L, Sai H, Dillmann WH, Hancock WW (2010). Inhibition of HDAC9 increases T regulatory cell function and prevents colitis in mice. Gastroenterology.

[8] de Zoeten EF (2011). Histone deacetylase 6 and heat shock protein 90 control the functions of Foxp3(+) T-regulatory cells. Mol. Cell Biol..

[9] Beier UH (2011). Sirtuin-1 targeting promotes Foxp3+ T-regulatory cell function and prolongs allograft survival. Mol. Cell Biol..

[10] Beier UH (2012). Histone deacetylases 6 and 9 and sirtuin-1 control Foxp3+ regulatory T cell function through shared and isoform-specific mechanisms. Sci. Signal..

[11] Beier UH (2015). Essential role of mitochondrial energy metabolism in Foxp3(+) T-regulatory cell function and allograft survival. FASEB J..

[12] Wang L (2015). FOXP3+ regulatory T cell development and function require histone/protein deacetylase 3. J. Clin. Invest..

[13] Xiao H (2016). HDAC5 controls the functions of Foxp3(+) T-regulatory and CD8(+) T cells. Int. J. Cancer.

[14] Hancock WW (2016). Isoform-Selective HDAC Inhibitor Therapy for Transplantation: Are We Ready for HDAC6?. Transplantation.

[15] Qian H (2017). HDAC6-mediated acetylation of lipid droplet-binding protein CIDEC regulates fat-induced lipid storage. J. Clin. Invest..

[16] Lieber AD (2019). Loss of HDAC6 alters gut microbiota and worsens obesity. FASEB J..

[17] Oehme I (2013). Histone deacetylase 10 promotes autophagy-mediated cell survival. Proc. Natl Acad. Sci. USA.

[18] Kao HY, Lee CH, Komarov A, Han CC, Evans RM (2002). Isolation and characterization of mammalian HDAC10, a novel histone deacetylase. J. Biol. Chem..

[19] Fischer DD (2002). Isolation and characterization of a novel class II histone deacetylase, HDAC10. J. Biol. Chem..

[20] Radhakrishnan R (2015). Histone deacetylase 10 regulates DNA mismatch repair and may involve the deacetylation of MutS homolog 2. J. Biol. Chem..

[21] Yang Y (2016). HDAC10 promotes lung cancer proliferation via AKT phosphorylation. Oncotarget.

[22] Wang B (2017). A Novel Role for Histone Deacetylase 10 (HDAC10) in the Regulation of PD-L1 Expression and Immune Tolerance Mediated By Antigen Presenting Cells (APCs). Blood.

[23] Liu Y (2013). Inhibition of p300 impairs Foxp3(+) T regulatory cell function and promotes antitumor immunity. Nat. Med..

[24] van Loosdregt J (2011). Rapid temporal control of Foxp3 protein degradation by sirtuin-1. PLoS One.

[25] Osada H (2004). Reduced expression of class II histone deacetylase genes is associated with poor prognosis in lung cancer patients. Int. J. Cancer.

[26] Elstrom RL (2004). Akt stimulates aerobic glycolysis in cancer cells. Cancer Res..

[27] Gerriets VA (2016). Foxp3 and Toll-like receptor signaling balance Treg cell anabolic metabolism for suppression. Nat. Immunol..

[28] Angelin A (2017). Foxp3 Reprograms T Cell Metabolism to Function in Low-Glucose, High-Lactate Environments. Cell Metab..

[29] Geraldy M (2019). Selective Inhibition of Histone Deacetylase 10: Hydrogen Bonding to the Gatekeeper Residue is Implicated. J. Med. Chem..

[30] Ibrahim Uba Abdullahi, Yelekçi Kemal (2019). Homology modeling of human histone deacetylase 10 and design of potential selective inhibitors. Journal of Biomolecular Structure and Dynamics.

[31] Hai Y, Shinsky SA, Porter NJ, Christianson DW (2017). Histone deacetylase 10 structure and molecular function as a polyamine deacetylase. Nat. Commun..

[32] Jin Z (2014). Decreased expression of histone deacetylase 10 predicts poor prognosis of gastric cancer patients. Int. J. Clin. Exp. Pathol..

[33] Oehme I, Lodrini M, Brady NR, Witt O (2013). Histone deacetylase 10-promoted autophagy as a druggable point of interference to improve the treatment response of advanced neuroblastomas. Autophagy.

[34] Ridinger J (2018). Dual role of HDAC10 in lysosomal exocytosis and DNA repair promotes neuroblastoma chemoresistance. Sci. Rep..

[35] Cicenas J (2008). The potential role of Akt phosphorylation in human cancers. Int. J. Biol. Markers.

[36] Tanaka A, Sakaguchi S (2017). Regulatory T cells in cancer immunotherapy. Cell Res..

[37] Song C, Zhu S, Wu C, Kang J (2013). Histone deacetylase (HDAC) 10 suppresses cervical cancer metastasis through inhibition of matrix metalloproteinase (MMP) 2 and 9 expression. J. Biol. Chem..

[38] Gialeli C, Theocharis AD, Karamanos NK (2011). Roles of matrix metalloproteinases in cancer progression and their pharmacological targeting. FEBS J..

[39] Skarnes WC (2011). A conditional knockout resource for the genome-wide study of mouse gene function. Nature.

[40] Akimova T (2014). Targeting sirtuin-1 alleviates experimental autoimmune colitis by induction of Foxp3+ T-regulatory cells. Mucosal Immunol..

