# Supplementary material for: HDAC10 deletion promotes Foxp3+ T-regulatory cell function
Source: Sci Rep. 2020 Jan 16;10:424. doi: 10.1038/s41598-019-57294-x (PMC6965082; doi:10.1038/s41598-019-57294-x)

Uncut blots to Figure 1a

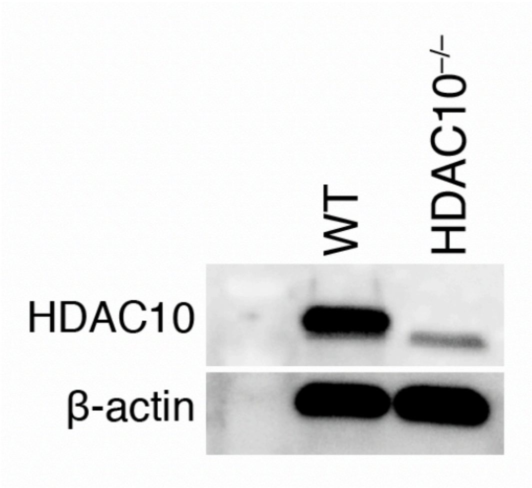

Fig. 1A

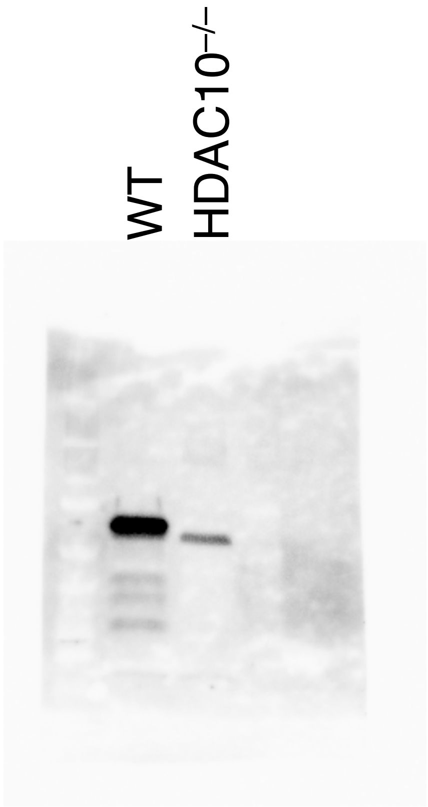

IB: HDAC10 (72 kDa)

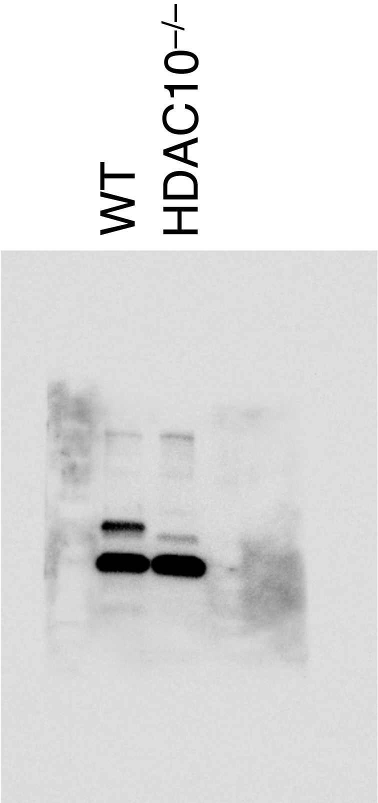

IB: Actin (42 kDa)
stripped/reblotted

Uncut blots to Figure 4d, upper half

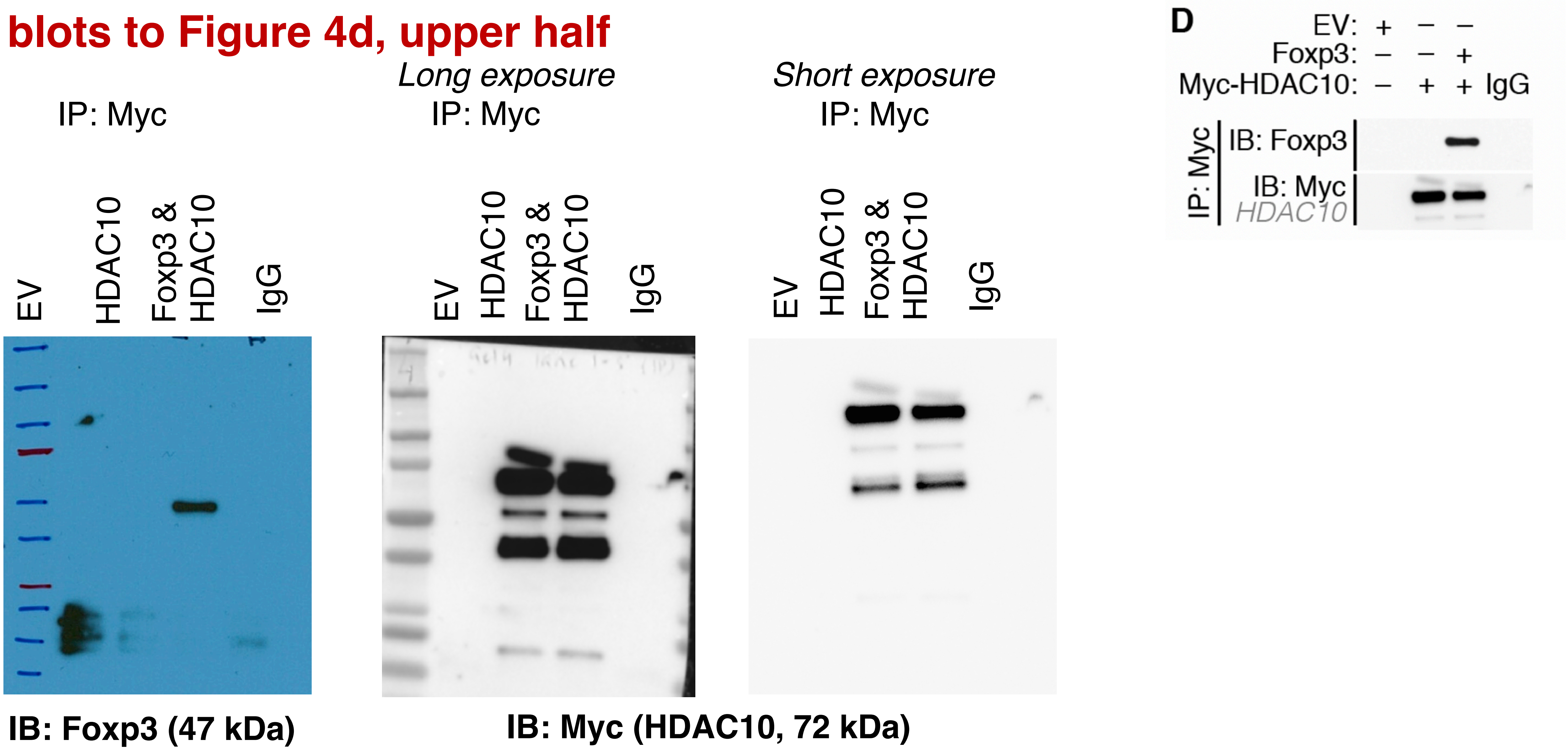

Uncut blots to Figure 4d, lower half

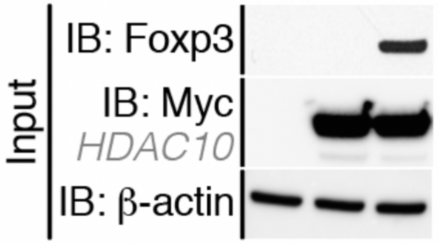

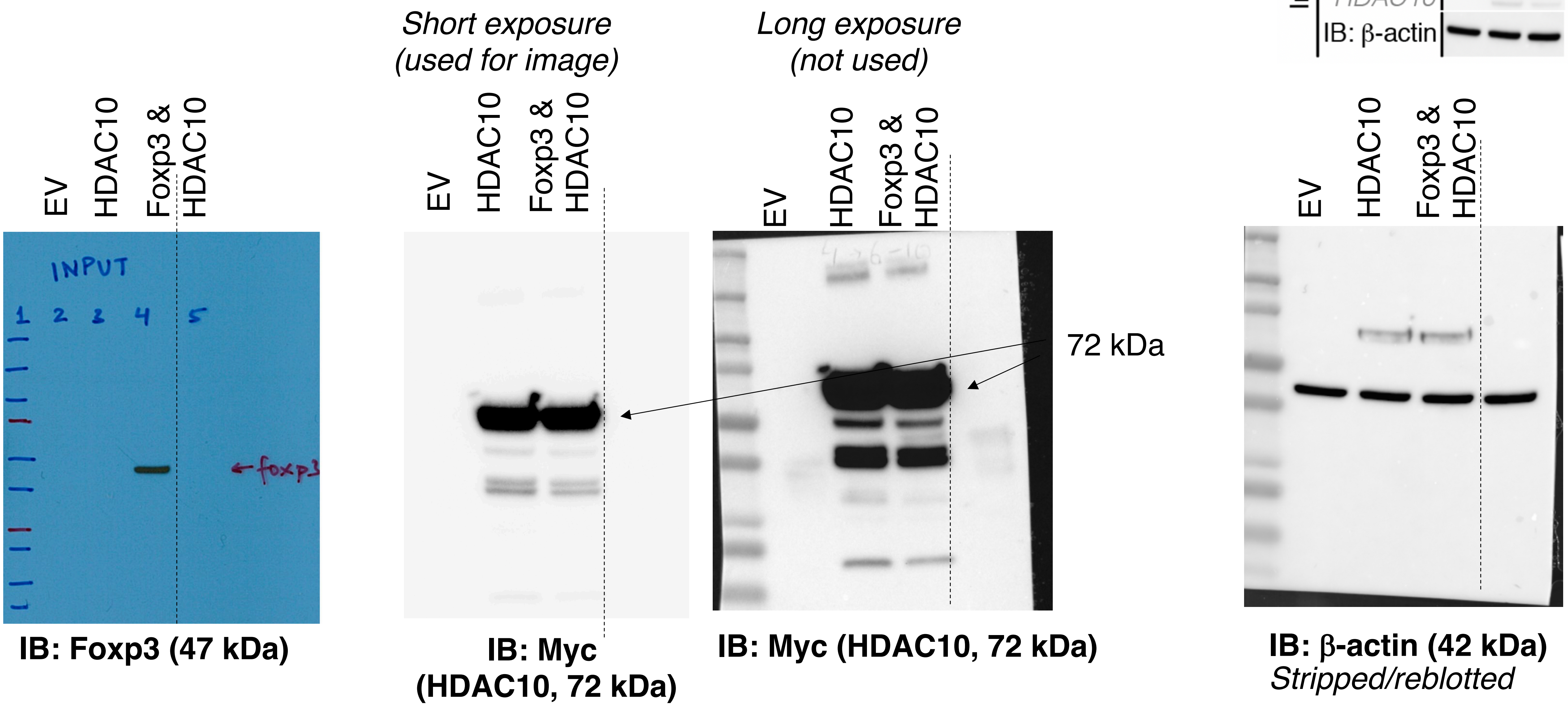

Uncut blots to Figure 4e (part 1, upper half)

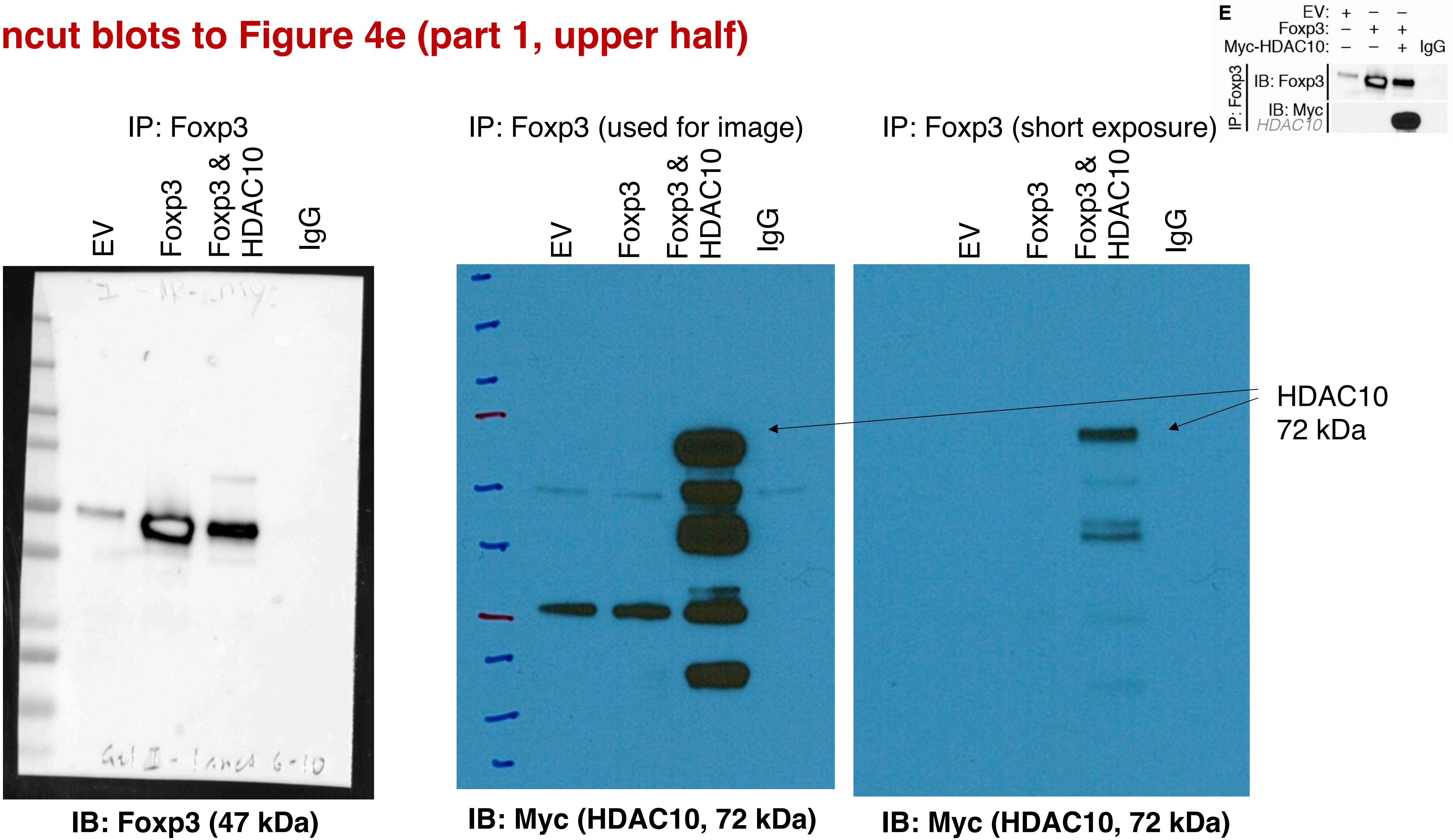

Uncut blots to Figure 4e (part 2, lower half)

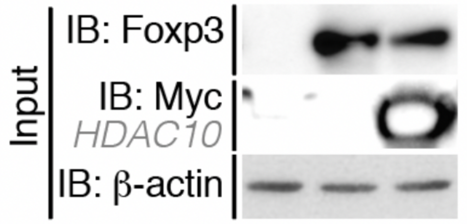

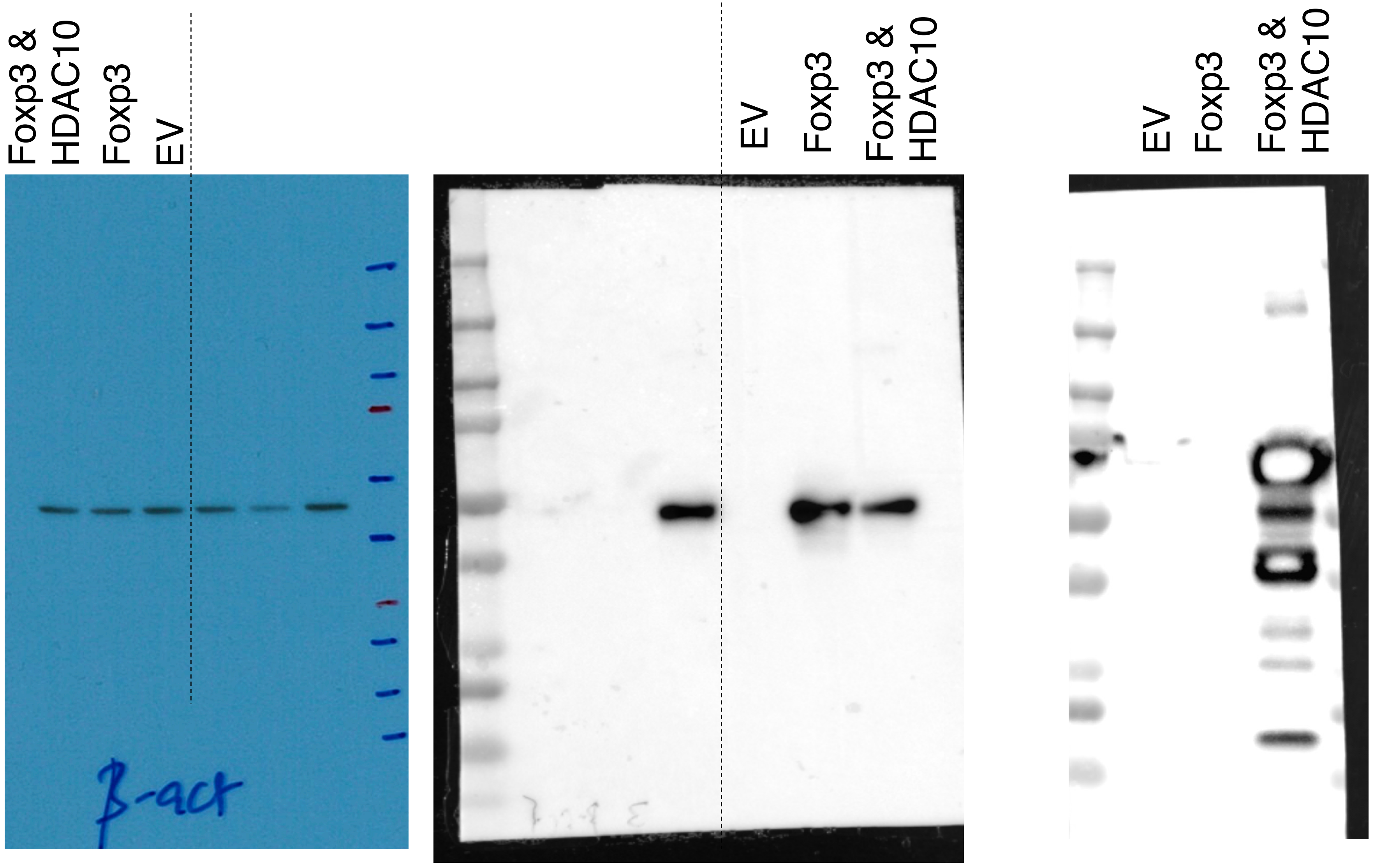

IB: β-actin (42 kDa)
Reversed orientation

IB: Foxp3 (47 kDa)

IB: Myc (HDAC10, 72 kDa)

Uncut blots to Figure 4f, part 1

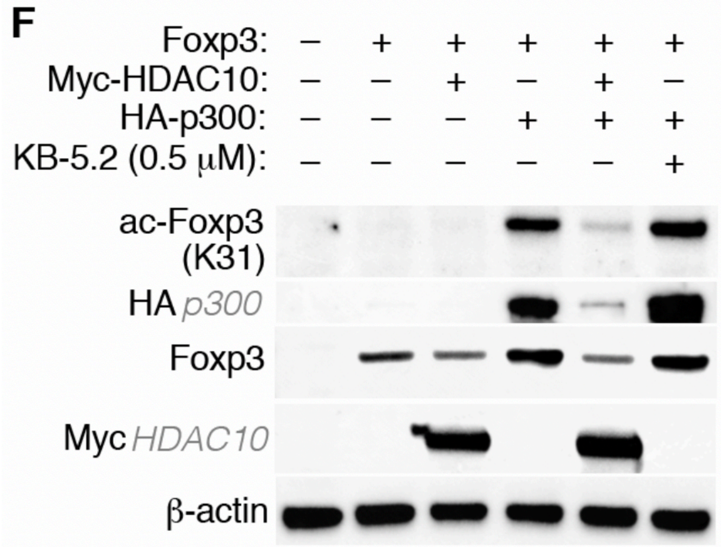

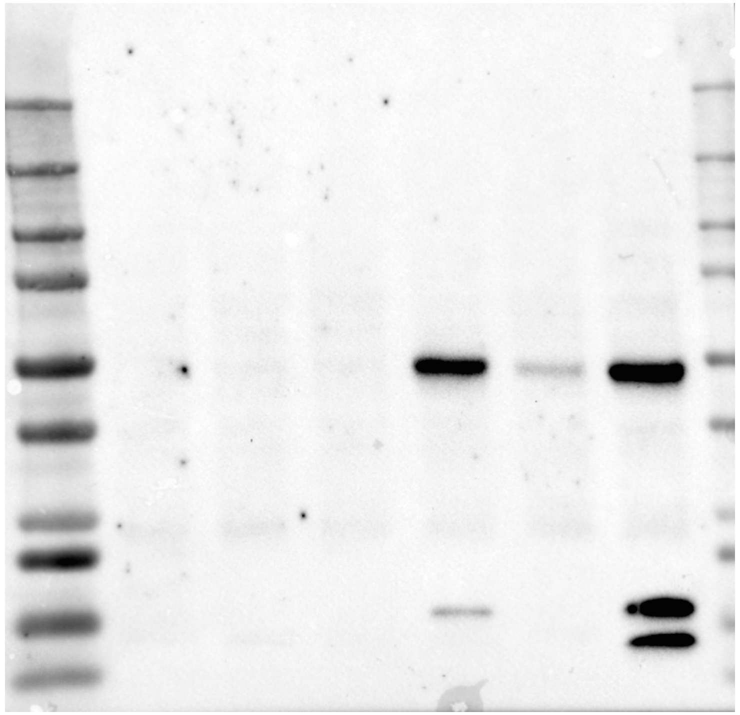

IB: Foxp3-K31ac (47 kDa)

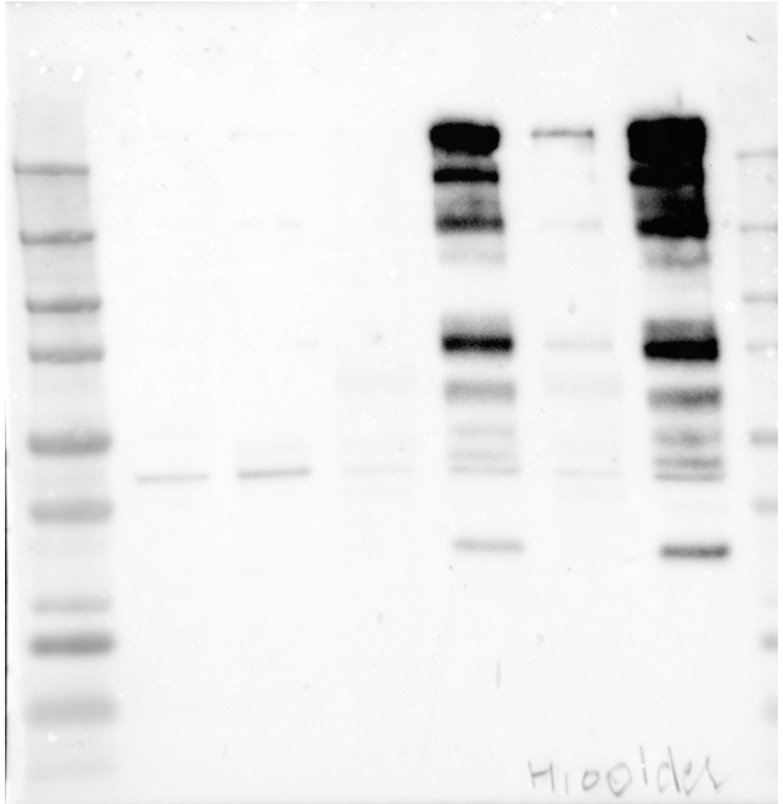

IB: HA (p300, 300 kDa)

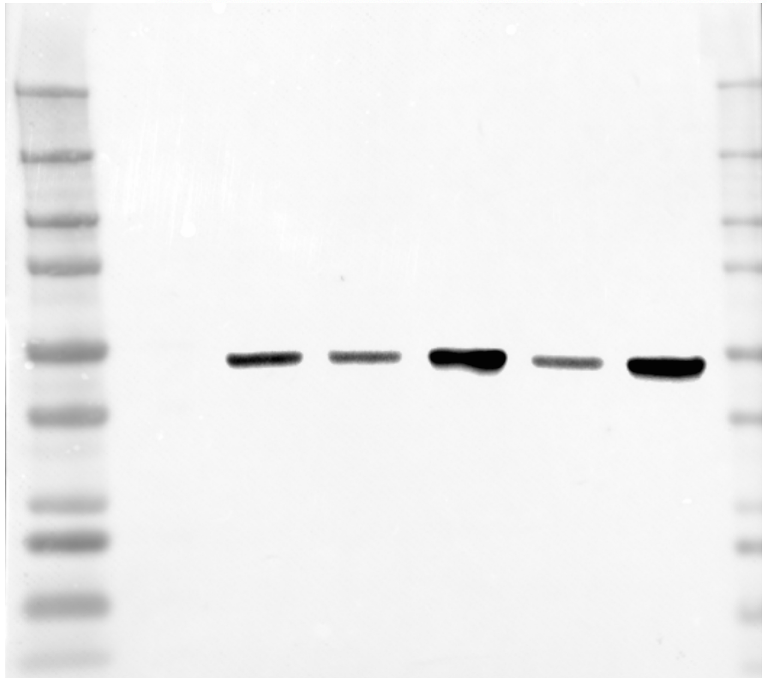

IB: Foxp3 (47 kDa)

Uncut blots to Figure 4f, part 2

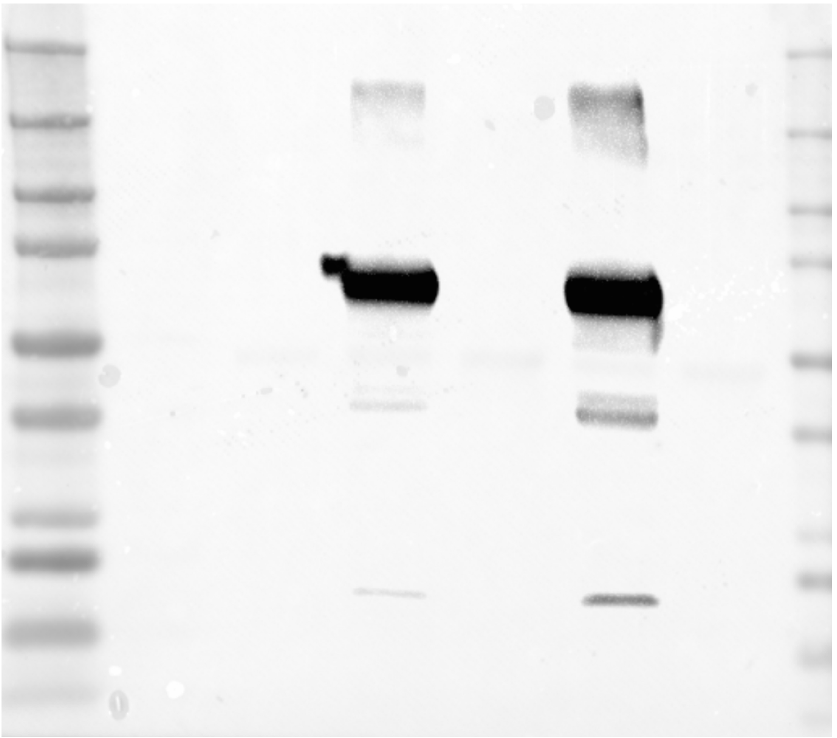

IB: Myc (HDAC10, 72 kDa)

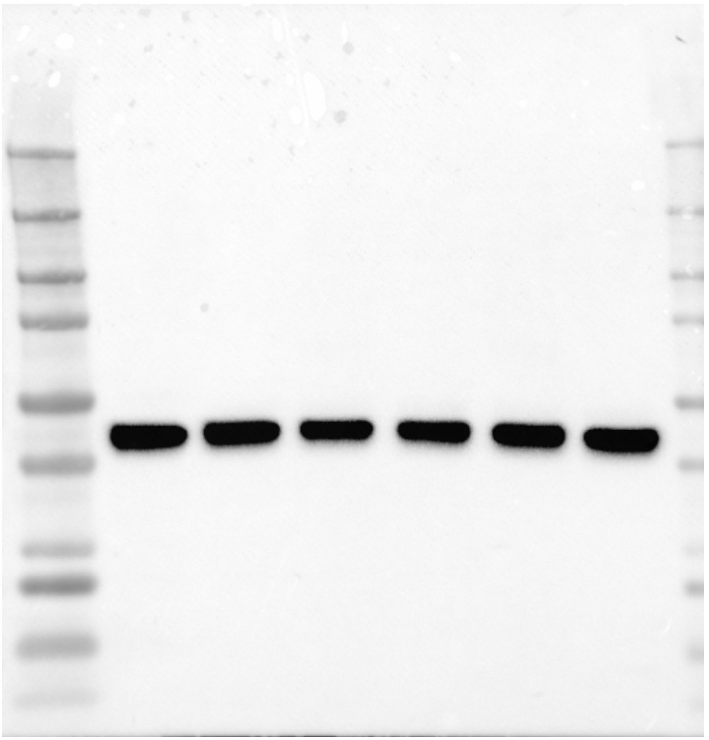

IB: β-actin (42 kDa)

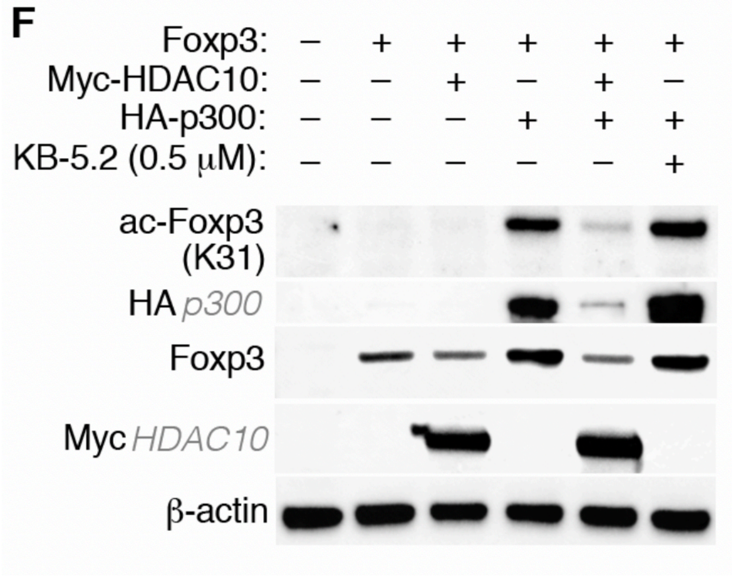

Uncut blots to Figure 4g

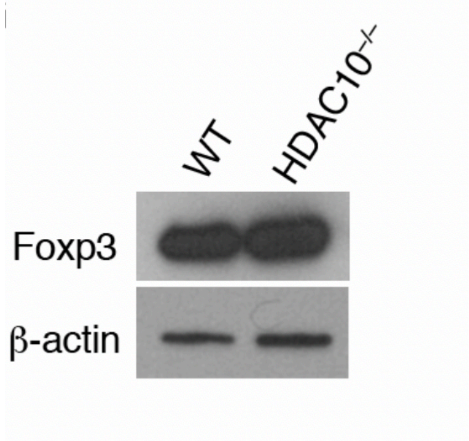

Greyscale image of film used to generate figure

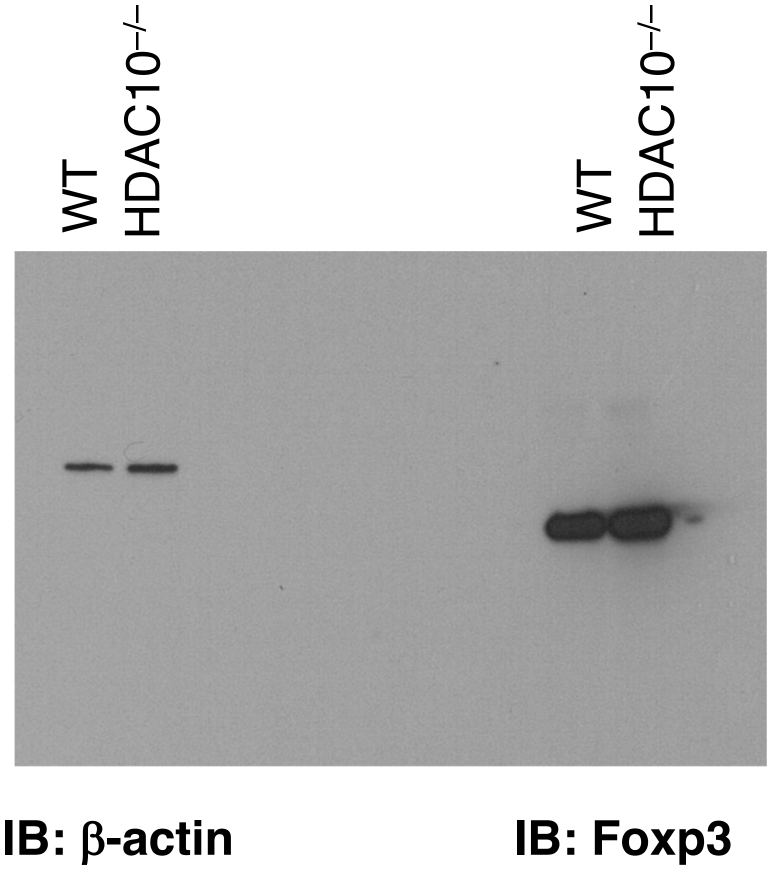

Color re-scan of film 2019

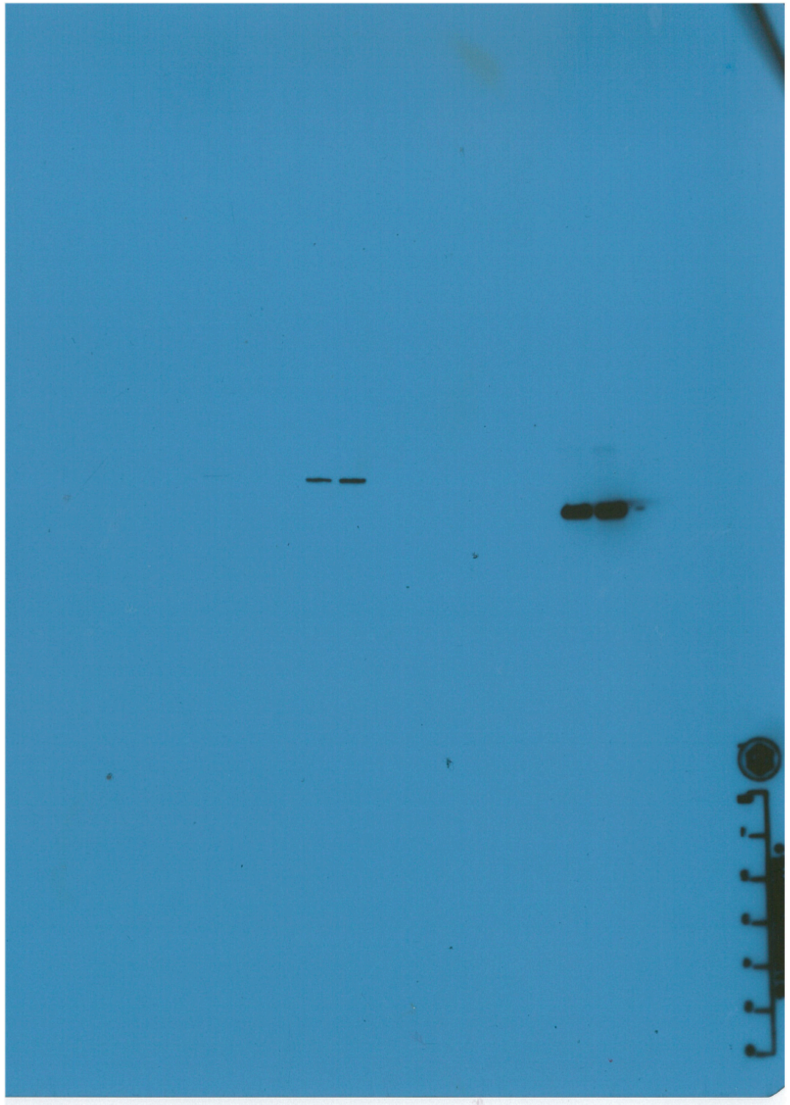

Supplement: Supplementary file 1 — Supplementary Dataset 1. [file 41598_2019_57294_MOESM1_ESM.pdf]
